# Generation of inositol polyphosphates through a phospholipase C-independent pathway involving carbohydrate and sphingolipid metabolism in *Trypanosoma cruzi*

**DOI:** 10.1128/mbio.03318-24

**Published:** 2025-04-02

**Authors:** Mayara S. Bertolini, Sabrina E. Cline, Miguel A. Chiurillo, Brian S. Mantilla, Aharon Eidex, Logan P. Crowe, Danye Qiu, Henning J. Jessen, Adolfo Saiardi, Roberto Docampo

**Affiliations:** 1 Department of Cellular Biology, Center for Tropical and Emerging Global Diseases, University of Georgia 200747, Athens, Georgia, USA; 2 Department of Biological Sciences, University of Cincinnati 118729, Cincinnati, Ohio, USA; 3 School of Chemistry, University of Leeds 4468, Leeds, England, United Kingdom; 4 Institute of Organic Chemistry & CIBSS-Centre for Integrative Biological Signalling Studies, University of Freiburg 9174, Freiburg, Baden-Württemberg, Germany; 5 Medical Research Council Laboratory for Cell Biology, University College London 4919, London, England, United Kingdom; Washington University in St. Louis School of Medicine, St. Louis, Missouri, USA; The Border Biomedical Research Center, El Paso, Texas, USA

**Keywords:** *Trypanosoma cruzi*, inositol pyrophosphates, phospholipase C, sphingolipids, inositol phosphoceramide

## Abstract

Inositol phosphates are involved in a myriad of biological roles and activities such as Ca^2+^ signaling, phosphate homeostasis, energy metabolism, and disease pathogenicity. In *Saccharomyces cerevisiae,* synthesis of inositol phosphates occurs through the phosphoinositide phospholipase C (PLC)-catalyzed hydrolysis of phosphatidylinositol 4,5-bisphosphate (PIP_2_) into inositol 1,4,5-trisphosphate (IP_3_) and diacylglycerol and further IP_3_ phosphorylation by additional kinases that leads to the formation of highly phosphorylated inositol derivatives, known as inositol pyrophosphates. Inositol-tetrakisphosphate 1-kinase (ITPK1) is an enzyme that mediates a PLC-independent inositol polyphosphate synthesis through phosphorylation of inositol monophosphates and other intermediates in the cytosol. In this work, we identified and characterized a *Trypanosoma cruzi* ITPK1 (TcITPK1) homolog. The ability of TcITPK1 to act as the mediator for this alternative pathway was established through *plc1*Δ and *plc1*Δ *isc1*Δ yeast complementation assays and SAX-HPLC analyses of radioactively labeled inositol. TcITPK1 localizes to the cytosol, and knockout attempts of *TcITPK1* revealed that only one allele was replaced by the DNA donor cassette at the specific locus, suggesting that *null* alleles may have lethal effects in epimastigotes. Ablation of *T. cruzi* phosphoinositide phospholipase C 1 (*TcPI-PLC1*) affected the synthesis of IP_3_ from glucose 6-phosphate but did not affect the synthesis of inositol polyphosphates, while ablation of inositol phosphosphingolipid phospholipase (*TcISC1*) affected the synthesis of inositol polyphosphates, thus revealing that the PLC-independent pathway using either glucose 6-phosphate or inositol phosphoceramide is involved in the synthesis of inositol polyphosphates, while the PLC-dependent pathway is involved in IP_3_ formation needed for Ca^2+^ signaling.

**IMPORTANCE:**

Millions of people are infected with *Trypanosoma cruzi,* and the current treatment is not satisfactory. Inositol pyrophosphates have been established as important signaling molecules. Our work demonstrates the presence of a phospholipase C-independent pathway for the synthesis of inositol pyrophosphates in *T. cruzi*. Furthermore, we demonstrate that this pathway starts with the synthesis of inositol monophosphates from glucose 6-phosphate or from inositol phosphoceramide, linking it to carbohydrate and sphingolipid metabolism. The essentiality of the pathway for the survival of *T. cruzi* infective stages makes it an ideal drug target for treating American trypanosomiasis.

## INTRODUCTION

*Trypanosoma cruzi* is an intracellular parasite and the causative agent for Chagas disease in humans. This is a zoonotic infection endemic to the Americas that currently affects 6–7 million individuals (1) and can infect many animal species. This parasite belongs to the eukaryotic supergroup Discoba, which is highly divergent from the Opisthokonta supergroup, which includes animals and fungi (2). Although some basic aspects of metabolism could be similar, each eukaryotic supergroup evolved independently and has unique characteristics that could be exploited for developing ways to eliminate the parasite without affecting its host. A case in point is the inositol phosphate pathway that is conserved in trypanosomes but with peculiarities not seen in animal cells.

**Fig 1:**
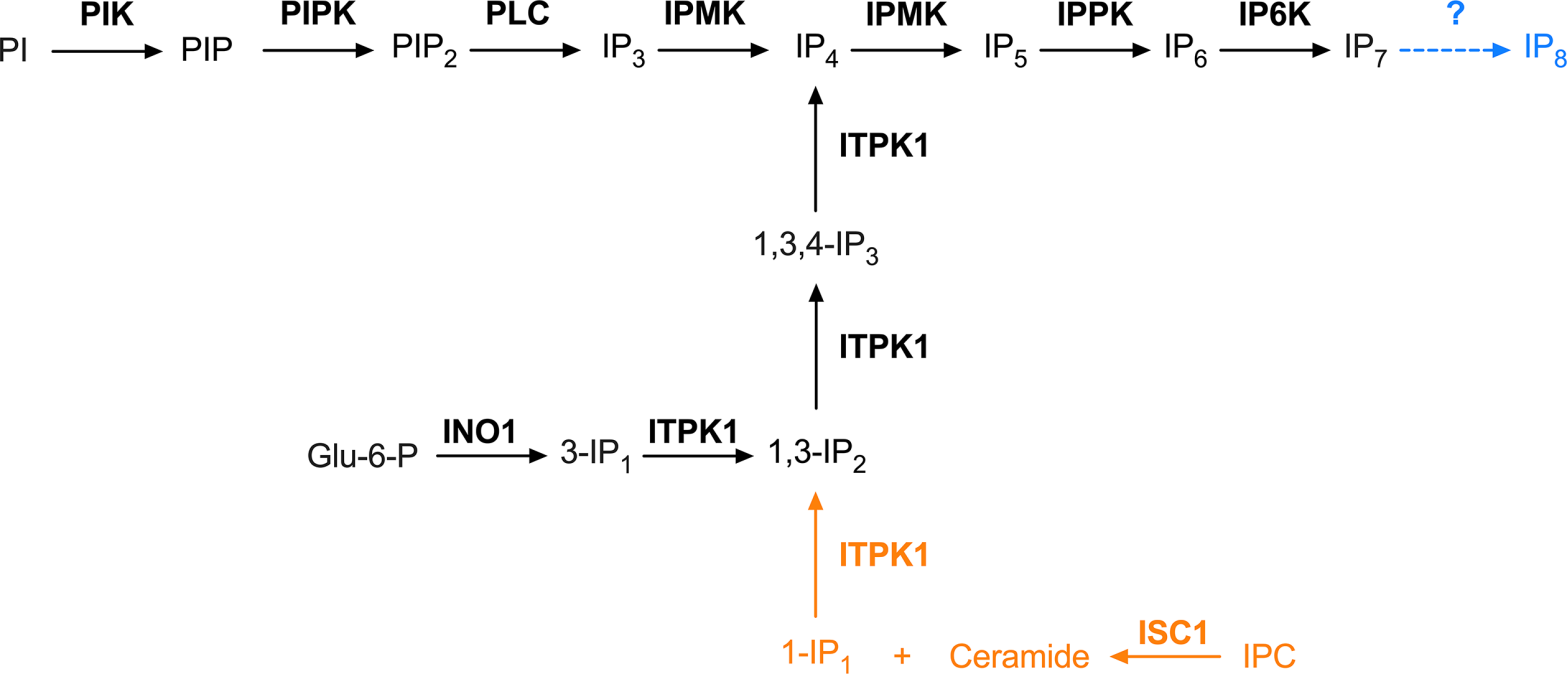
Synthesis of inositol pyrophosphates. Phosphatidylinositol (PI) is phosphorylated by a PI kinase (PIK) to form phosphatidylinositol phosphate (PI-4-P or PIP), and a PIP kinase (PIPK) generates phosphatidylinositol 4,5-bisphosphate (PIP_2_). A phospholipase C (PLC) catalyzes the formation of inositol 1,4,5-trisphosphate (IP_3_) from PIP_2_ via the lipid route. IP_3_ is further converted into inositol polyphosphates through kinase reactions catalyzed by inositol phosphate multikinase (IPMK), which forms inositol tetrakisphosphate (IP_4_) and inositol pentakisphosphate (IP_5_); inositol pentakisphosphate kinase (IPPK), which forms inositol hexakisphosphate (IP_6_); and inositol hexakisphosphate kinase (IP6K), which forms diphosphoinositol pentakisphosphate (IP_7_) and possibly bis-diphosphoinositol tetrakisphosphate (IP_8_). Alternative routes (cytosolic routes) start with the conversion of glucose 6-phosphate to inositol 3-phosphate (3-IP_1_) by inositol-3-phosphate synthase (INO1), followed by kinase reactions catalyzed by inositol tetrakisphosphate 1-kinase (ITPK1), or start with the formation of inositol 1-phosphate (1-IP_1_) from inositol phosphoceramide (IPC) by inositol sphingolipid phospholipase C-like protein (ISCL), with further kinase reactions by ITPK1. Note that PLC produces inositol 1,4,5-trisphosphate, while the alternative pathways likely produce inositol 1,3,4-trisphosphate. The enzyme that catalyzes the conversion of IP_7_ into IP_8_ (blue) in *T. cruzi* is unknown. The pathway that converts IPC into 1-P_1_ and ceramide (orange) is present in *T. cruzi* but absent in mammals.

Inositol phosphates regulate a large number of cellular functions such as Ca^2+^ signaling, energy metabolism, and phosphate homeostasis (3). The precursor of inositol phosphates in trypanosomes, *myo-*inositol, can be acquired from the extracellular medium *via* an inositol symporter (4–7) or synthesized endogenously (8, 9). Another potential source is by salvage of lipids from the host cells and remodeling in the mammalian stages, as occurs in *Leishmania* spp. (10, 11). Once inside the cells, inositol combines with CDP-diacylglycerol to form phosphatidylinositol (PI) in a reaction catalyzed by a phosphatidylinositol synthase (PIS). PI is phosphorylated by a PI kinase to form phosphatidylinositol phosphate (PI-4-P or PIP), and a PIP kinase (PIPK) generates phosphatidylinositol 4,5-bisphosphate (PIP_2_) (Fig. 1). The route for synthesis of inositol phosphates described in budding yeast (*S. cerevisiae*) involves the action of a phospholipase C (PLC) on PIP_2_ releasing inositol 1,4,5-trisphosphate (IP_3_) and diacylglycerol (12) (Fig. 1). IP_3_ has a well-studied receptor, the IP_3_ receptor, which participates in Ca^2+^ release from intracellular stores such as the endoplasmic reticulum (ER) in many eukaryotes (13). However, this receptor is found in acidocalcisomes of trypanosomes (14, 15). IP_3_ can be further phosphorylated in mammals, trypanosomes, and *S. cerevisiae* at different hydroxyl positions producing IP_4_ and IP_5_ by inositol phosphate multikinase (IPMK; known as Arg82 in budding yeast) and IP_6_ by inositol pentakisphosphate kinase (IPPK; known as Ipk1 in yeast), producing the fully phosphorylated form known as inositol hexakisphosphate (IP_6_), or phytic acid (Fig. 1). IP_5_ and IP_6_ can be the precursors of diphosphoinositol phosphates (PP-IPs, or simply IP_7_, IP_8_), also called inositol pyrophosphates (16). Inositol pyrophosphates are characterized by the presence of single (PP-IP_4_ and PP-IP_5_) or double (PP_2_-IP_3_ and PP_2_-IP_4_) pyrophosphate moieties linked at different positions of the *myo*-inositol core (17). In trypanosomes, the biosynthesis of IP_7_ takes place by the action of inositol hexakisphosphate kinase (IP6K; known as Kcs1 in yeast) using IP_6_ as the substrate and ATP as the phosphate donor (18, 19) (Fig. 1). Our molecular and physiological studies done with the phosphate/sodium symporters of *T. brucei* acidocalcisomes (TbPho91) and yeast vacuoles (Pho91p) demonstrated that IP_7_ interacts with their SPX domains, and this is essential for phosphate release to the cytosol (20). Therefore, IP_7_ has been proposed as a signaling molecule in trypanosomes with critical importance in phosphate homeostasis.

In mammalian cells (21), *S. cerevisiae* (22), and trypanosomes (6), in addition to its uptake from the extracellular medium, *myo*-inositol can be generated endogenously by isomerization of glucose 6-phosphate (G6P) into inositol 3-phosphate (3-IP_1_), catalyzed by the inositol 3-phosphate synthase (known as INO1 in yeast and ISYNA1 in mammals). In mammalian cells, 3-IP_1_ can be further phosphorylated by the inositol tetrakisphosphate 1-kinase 1 (ITPK1) (22, 23) to produce substrates for other inositol phosphate kinases such as IPMK and IPPK and generate IP_6_ and inositol pyrophosphates (Fig. 1). An alternative PLC-independent pathway discovered in yeast PLC *null* mutants transformed with *HsITPK1* could potentially utilize endogenous inositol generated from inositolphosphoceramide (IPC) (22), a highly abundant sphingolipid in trypanosomes, fungi, and plants but absent in mammals, as a substrate for ITPK1 (Fig. 1). In mammalian cells, ISYNA1 knockout cells are still capable of producing inositol polyphosphates, indicating the existence of an alternative endogenous pathway for their synthesis (24).

In this work, we investigated the role of the PLC-dependent and PLC-independent pathways in the synthesis of inositol pyrophosphates in *T. cruzi*. Knockout of *T. cruzi* phosphoinositide phospholipase C 1 (*TcPI-PLC1*) revealed that the PLC-dependent pathway is not necessary for the synthesis of inositol polyphosphates, while TcITPK1 is required for their PLC-independent synthesis, using either glucose 6-phosphate or inositolphosphoceramide as a source of inositol monophosphate.

## RESULTS

### Characterization of TcITPK1

One gene (TcYC6_0083580; KAF8297109.1) encoding a putative inositol tetrakisphosphate 1-kinase 1 (TcITPK1) was found in the *T. cruzi* Y strain genome at the TriTryp and NCBI databases. The orthologs identified in the related trypanosomatids *T. brucei* (Tb927.8.290; XP_847459) and *Leishmania major* (LmjF.24.1930; XP_001683711.1) shared 39.7% and 21.9% amino acid identity, respectively, to TcITPK1, which shares 17.1% identity and 30.7% similarity with human ITPK1 (NP_001136065). The open reading frame predicts a protein of 419 amino acids with a molecular weight of 46.58 kDa.

ITPK1 is a conserved inositol phosphate kinase. Recent studies have demonstrated that *Lokiarchaeum candidatum* ITPK1 shares structural and homology similarities with *Homo sapiens* and *Entamoeba histolytica* ITPK1. Highly conserved residues at H167, K199, and R212 of HsITPK1 are located near the IP-contact residues and are proximal to the ATP-binding site (22). Additionally, these ITPK1 homologs share less conserved residues at K18, K59, H162, and G301 (22). A Clustal Omega multiple sequence alignment of previously established homologs, including sequences derived from the kingdoms Plantae (*Oryza sativa* and *Zea mays*), Animalia (*Homo sapiens, Mus musculus, Gallus gallus,* and *Danio rerio*), Protista (*Dictyostelium discoideum*, *Entamoeba histolytica,* and *Monosiga brevicollis*), and Archaea (*Lokiarchaeum candidatus*) with the hypothetical protein found in *T. cruzi,* was completed to determine if the protein TcYC6_0083580 has the conserved inositol phosphate and ATP contact residues. The *T. cruzi* hypothetical protein was shown to share the H167 and K199 highly conserved HsITPK1 residues (H198 and K242 in TcITPK1) and a few of the less conserved HsITPK1 residues at K18, K59, and G301 (K20, K77, and G378 in TcITPK1) (Fig. S1). The CLUSTAL Omega alignment reinforces the hypothesis that the hypothetical protein TcYC6_0083580 is TcITPK1.

Once TcYC6_0083580 was established as a TcITPK1 ortholog, a phylogenetic tree was constructed to investigate the evolutionary relationships of the ITPK1 gene in kinetoplastid parasites and, by association, the PLC-independent inositol phosphate pathway (Fig. S2). The tree included several outgroup species, such as *Lokiarchaeum candidatus*, *Naegleria fowleri*, *Entamoeba histolytica*, and *Homo sapiens*, which were used to provide a clear evolutionary baseline for comparison with the kinetoplastid parasites. Within the kinetoplastid parasite branches, there is a strong evolutionary clustering of ITPK1, with most branches having bootstrap values above 80, indicating high statistical support for the relationships.

**Fig 2:**
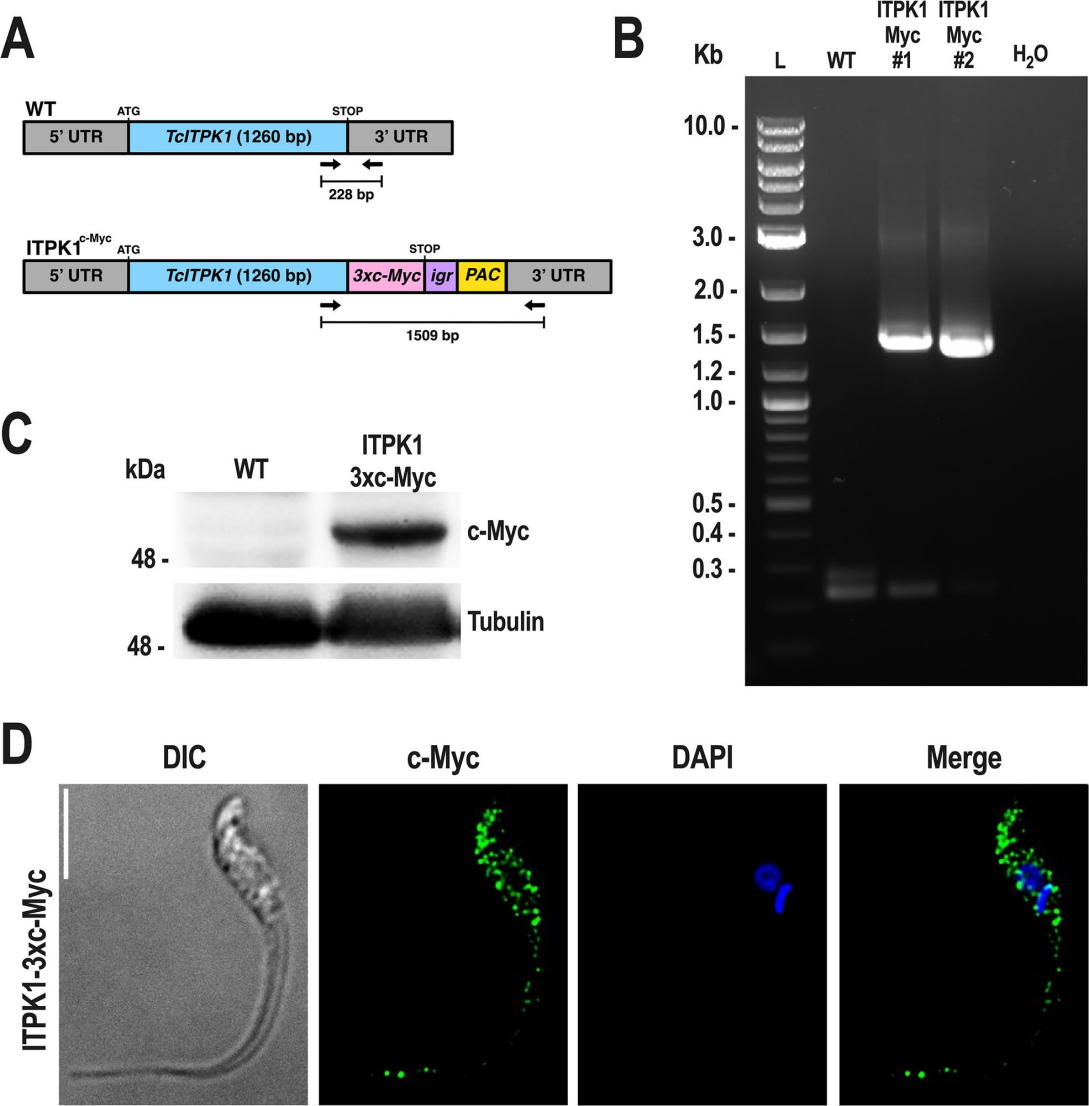
CRISPR/Cas9 endogenous C-terminal tagging of *TcITPK1*. (A) Schematic representation of the wild-type and endogenous tagged *TcITPK1* gene. Epimastigotes were endogenously tagged with a 3×c-Myc tag using CRISPR/Cas9 genome editing. (B) PCR analysis for validation of *TcITPK1* tagging showing expected bands for control cell lines (predicted size, 228 bp) and *TcITPK1*-3×c-Myc cell line (predicted size, 1,509 bp). Lanes: L, 1 kb plus ladder; WT, wild-type; ITPK Myc#1, *TcITPK1*-3×c-Myc#1; ITPKMyc#2, *TcITPK1*-3×c-Myc #2; H_2_O, PCR negative control. Clone#1 was selected for further study as clone#2 was smaller. (C) Western blot analysis of wild-type and TcITPK1-3×c-Myc epimastigotes using the monoclonal antibody against c-Myc tag. The predicted protein molecular mass for TcITPK1-3×c-Myc is 50.9 kDa. Molecular markers are on the left. Tubulin was used as a loading control. (D) Localization of endogenously tagged TcITPK1-3×c-Myc in epimastigotes using anti-c-Myc antibodies. DIC, differential interference contrast. c-Myc (green), TcITPK1-3×c-Myc. The merge image shows TcITPK1-3×c-Myc (green) and DAPI staining (blue). Scale bar = 5  µm.

To define the cellular localization of TcITPK1, CRISPR/Cas9 endogenous C-terminal tagging of TcITPK1 with a 3×c-Myc tag was done (Fig. 2A) and verified by PCR (Fig. 2B shows predominant bands of 1,500 bp) and Western blot analysis (Fig. 2C). Immunofluorescence assays in epimastigotes localized this protein to the cytosol of the parasite (Fig. 2D).

AlphaFold-2.1.1 (25) was utilized to predict the structural conformation of TcITPK1 and HsITPK1. Both the relaxed (Fig. S3A and C) and unrelaxed (Fig. S3B and D) ribbon models demonstrate high structural confidence in the core of the proteins near the inositol phosphate-contact and ATP-binding site residues with per-residue model confidence score (pLDDT) values of greater than 90, denoted in dark blue. There are some lower confidence regions of the TcITPK1 protein structure (denoted by yellow and orange); however, this lower confidence score may be due to evolutionary diversity outside of ITPK1’s catalytic site.

After three-dimensional resolution of TcITPK1 by AlphaFold-2.1.1, the model was analyzed by COFACTOR (26, 27) to glean structural-based function predictions for ligand-binding partners and ligand-binding sites. COFACTOR predicted that IP_3_ would bind to TcITPK1 at residues K20, T192, G193, H198, K242, Y244, Q277, N374, P377, and G378 (Fig. S3E). Additionally, the predicted TcITPK1 model with IP_3_ was structurally aligned with the HsITPK1 model using TM-align parameters on RCSB PDB (Fig. S3F).

The AlphaFold-2.1.1-predicted TcITPK1 model was also compared to the X-ray diffracted HsITPK1 (PDB: 2ODT) using the pairwise structural alignment tool (28–35). Both ITPK1 proteins had highly conserved secondary structures and minor spatial modulation of alpha-helical structures (Fig. S3G). Additionally, when comparing TcITPK1 with HsITPK1 (PDB: 2ODT), the two structures were found to have 272 equivalent positions with a root mean square deviation (RMSD) of 3.05 Å without twists, denoting strong alignment between the eukaryotic orthologous ITPK1 structures. The 0.57 TM-score demonstrated a similar protein structure between AlphaFold-2.1.1-TcITPK1 and HsITPK1 (PDB: 2ODT). Further structural studies are warranted.

### Functional validation *in vivo* of the role of TcITPK1 in the synthesis of inositol polyphosphates

We investigated the activity of *T. cruzi* ITPK1 *in vivo* by transforming *S. cerevisiae* strains with different genetic backgrounds: wild-type (BY4741), *PLC1*-ablated (*plc1*Δ), and *PLC1-* and *ISC1*-ablated (*plc1*Δ *isc1*Δ). The goal was to assess the production of IP_6_ (the most abundant inositol polyphosphate), IP_7_, and IP_8_. The *plc1*Δ strain was used to prevent IP_6_ formation through the yeast PLC1 pathway as PLC1 is the only pathway in yeast responsible for IP_6_ synthesis (22). In addition, it has been reported that in yeasts, degradation of IPC by inositol phosphosphingolipid phospholipase (known as ISC1 in yeast) generates ceramide and inositol 1-phosphate (1-IP_1_) (Fig. 1). 1-IP_1_ can be a substrate for HsITPK1 in yeasts lacking PLC1 (22). Human ITPK1 was used as a positive control, while strains transformed with an empty vector served as negative controls. We also included two TcITPK1 mutants, H198A and K242A, both of which are predicted to disrupt its activity, for transformation. Successful plasmid incorporation and protein expression were confirmed by PCR (Fig. S4A) and immunofluorescence assays, respectively (Fig. S4B).

**Fig 3:**
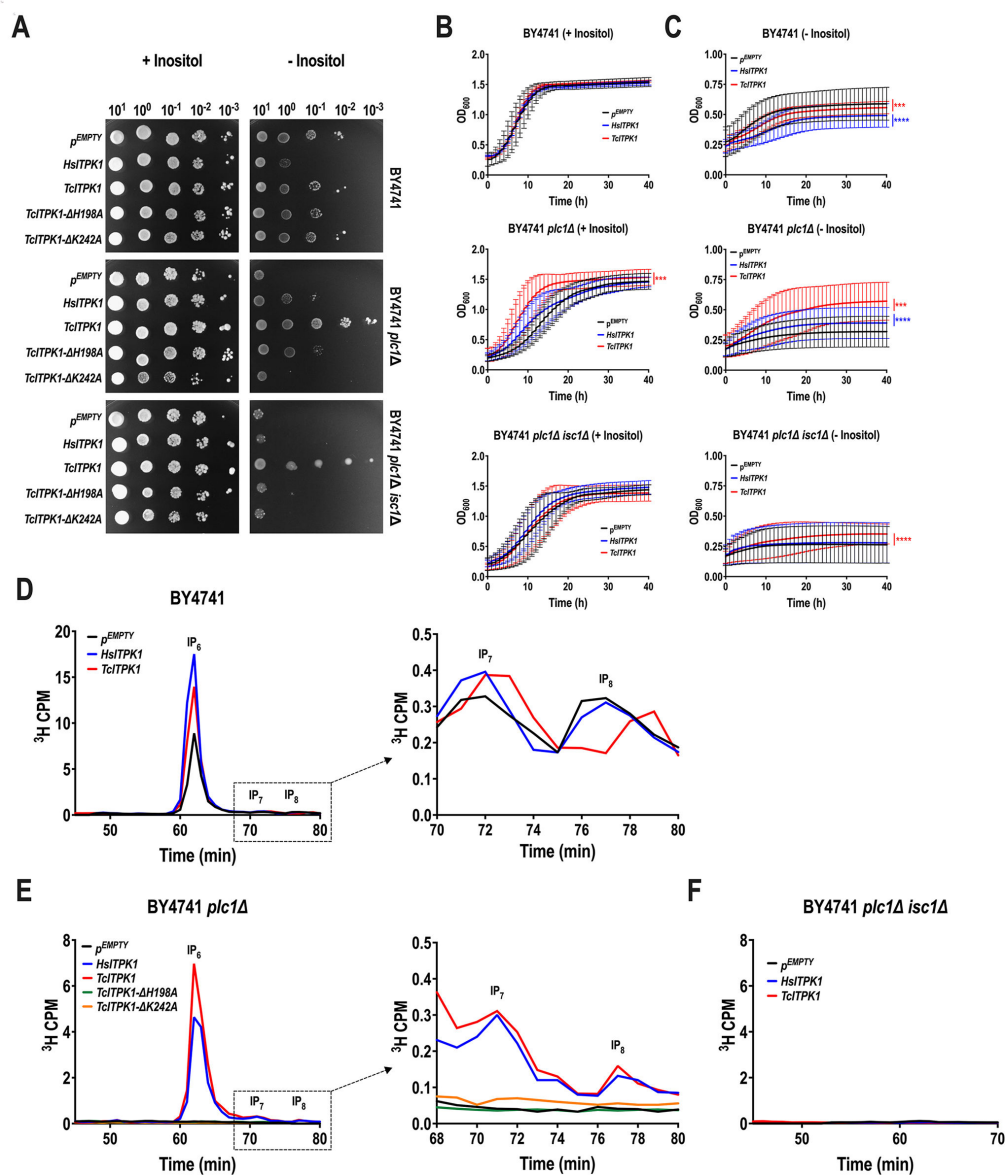
*TcITPK1* or *HsITPK* complementation in yeast. (A) Growth of *S. cerevisiae* wild-type (BY4741), *PLC1*-ablated (*plc1*Δ), and *PLC1-* and *ISC1*-ablated (*plc1*Δ *isc1*Δ) transformed with the empty vector pCA45 (pEMPTY), pCA45-*HsITPK1*, pCA45-*TcITPK1,* pCA45-*TcITPK1-H198A,* or pCA45-*TcITPK1-K242A* plasmids. Yeast cultures were adjusted to OD_600_ = 10 and spotted onto the SC-URA solid medium with or without *myo-*inositol along with four 10-fold serial dilutions. (B and C) Growth of *S. cerevisiae* wild-type (BY4741), *PLC1*-ablated (*plc1*Δ), and *PLC1-* and *ISC1*-ablated (*plc1*Δ *isc1*Δ) transformed with pCA45 (pEMPTY), pCA45-*HsITPK1,* or pCA45-*TcITPK1* in liquid SC-URA medium with (B) or without (C) *myo-*inositol starting at an OD_600_ of 0.1 was monitored every 30 minutes for a total period of 40 hours. Values are expressed as means ± S.D. (*n* = 3). ***P ≤ 0.001 and ****P ≤ 0.0001 by one-way ANOVA with Tukey’s multiple comparisons test. Blue asterisks = pEMPTY vs *HsITPK1*; red asterisks = pEMPTY vs *TcITPK1*. (D) SAX-HPLC analysis of IPs from the [^3^H]inositol-labeled wild-type yeast strain transformed with pEMPTY (pCA45, black line), pCA45-*HsITPK1* (blue line), or pCA45-*TcITPK1* (red line). (E) SAX-HPLC analysis of IPs from the [^3^H]inositol-labeled *PLC1*-ablated (*plc1*Δ) yeast strain transformed with pEMPTY (pCA45, black line), pCA45-*HsITPK1* (blue line), pCA45-*TcITPK1* (red line), pCA45-*TcITPK1-H198A* (green line), or pCA45-*TcITPK1-K242A* (yellow line). (F) SAX-HPLC analysis of IPs from the [^3^H]inositol-labeled *PLC1-* and *ISC1*-ablated (*plc1*Δ *isc1*Δ) yeast strains transformed with pEMPTY (pCA45, black line), pCA45-*HsITPK1* (blue line), or pCA45-*TcITPK1* (red line).

We tested the growth of these strains on normal SC-URA medium or medium without inositol (Fig. 3A) and found that the growth of all strains in the medium with inositol was normal regardless of whether they were transformed with the empty plasmid, HsITPK1, or TcITPK1. This indicates that external inositol compensates for any disruptions in inositol phosphate metabolism caused by the transformations. In contrast, on agar plates without inositol, the growth patterns varied. The wild-type strain exhibited impaired-to-partially normal growth when transformed with either HsITPK1 or TcITPK1, suggesting that these kinases may disrupt inositol phosphate metabolism under inositol-depleted conditions. The *plc1*Δ strain showed impaired growth with the empty plasmid, but growth was restored to partially normal levels with TcITPK1, indicating a compensatory effect by this kinase. Conversely, the *plc1*Δ *isc1*Δ strain displayed generally impaired growth across all conditions, although TcITPK1 provided some improvement. These results suggest that inositol phosphate metabolism is significantly affected by the absence of PLC1 and ISC1, with TcITPK1 offering compensation in the absence of inositol, whereas HsITPK1 does not alleviate the growth impairment as effectively. We also tested the growth of these strains in liquid SC-URA media with and without inositol, and the results were consistent with those observed on agar plates (Fig. 3B and C).

We assessed the impacts of ITPK1 complementation using SAX-HPLC analysis with [^3^H]inositol labeling to quantify the formation of inositol phosphate species. Both *TcITPK1* and *HsITPK1* could complement yeast deficient in IP_6_ formation, functionally validating the activity of these enzymes *in vivo* (Fig. 3D and E). Interestingly, both human and *T. cruzi* ITPK1 increased (above normal) the level of IP_6_ in the wild-type background (Fig. 3D). When TcITPK1 mutated in amino acids H198A and K242A, predicted to be part of the catalytic site, was used for transformation of *plc1*Δ yeast, no rescue was observed (Fig. 3E). In contrast to the results obtained in yeast *plc1*Δ mutants, in the *plc1*Δ *isc1*Δ strain, the level of IP_6_ was not rescued by either human or TcITPK1 (Fig. 3F), demonstrating that inositol derived from sphingolipids could be used by both TcITPK1 and HsITPK1 to generate IP_6_.

### Attempts to knockout *TcITPK1* gene expression

We designed a CRISPR/Cas9 strategy to generate KO mutants of this gene. The method involves the constitutive expression of Cas9 and specific single-guide RNA (sgRNA) and the utilization of a DNA donor template to promote double-strand break repair by homologous-directed repair (36). Two different cassettes were utilized along with two unique *TcITPK1*-targeted sgRNA designs. Each of the four unique designs was used in technical duplicates, and the complete co-transfection experiments were repeated a total of three independent times. Each of the transfections resulted in mixed populations of *TcITPK1-*ablated and WT epimastigotes. However, after limiting dilution to isolate *TcITPK1*-KO parasites, no epimastigotes survived the selection process, suggesting the essentiality of this gene for cell proliferation.

**Fig 4:**
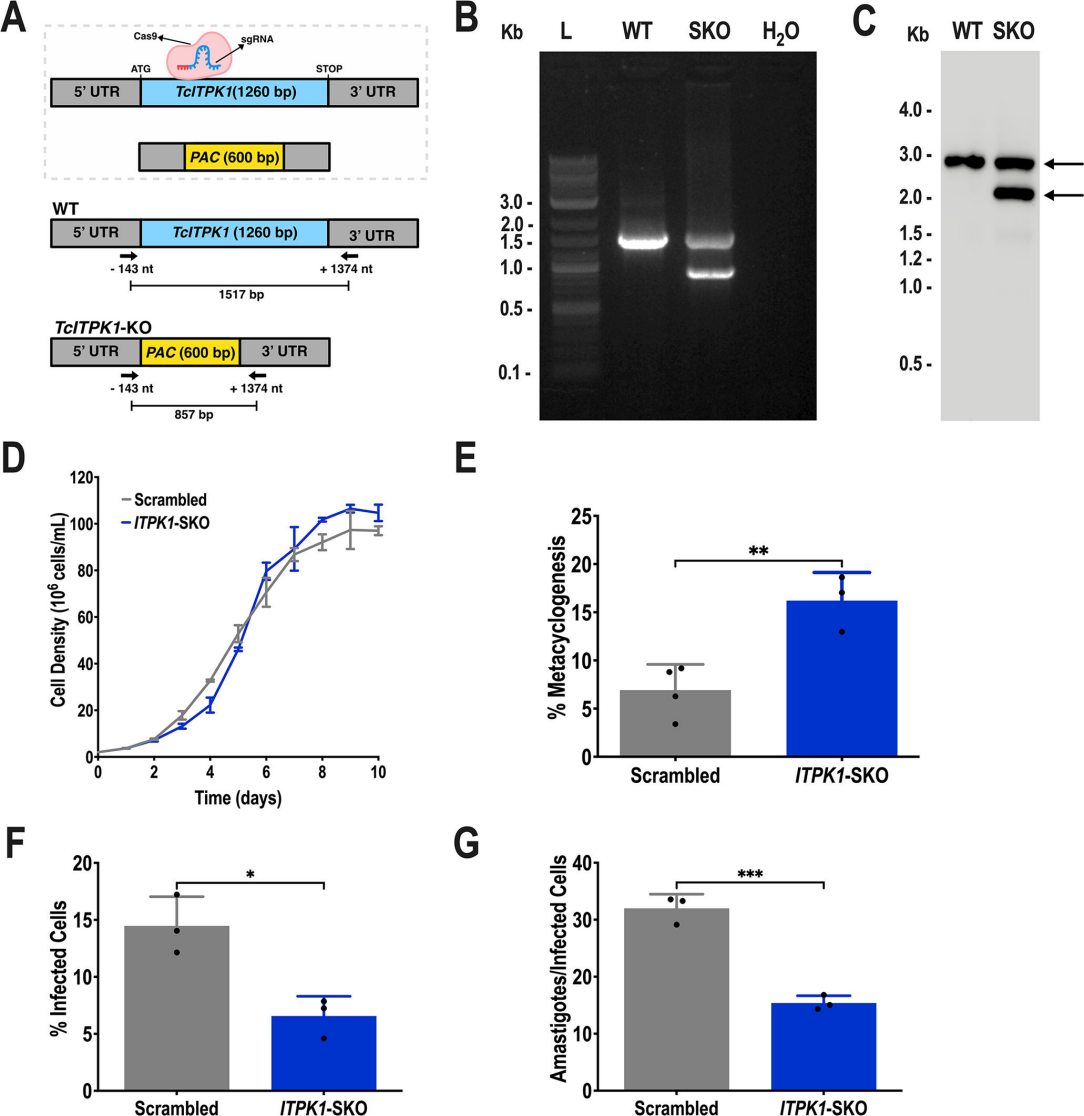
Single-gene knockout of *TcITPK1*. (A) Schematic representation of the strategy used to generate a *TcITPK1*-SKO mutant by homologous recombination and primers (arrows) used to verify gene replacement by PCR. The intact locus generates a PCR product of 1,517 bp, while the disrupted locus generates a fragment of 857  bp. (B) PCR analysis showing that a single gene of *TcITPK1* was ablated at its genomic locus and replaced in genomic DNA of the SKO cell line. Lanes: L, 1 kb plus ladder; WT, wild type; SKO, *TcITPK1*-SKO; H_2_O, PCR negative control. (C) Southern blot analysis of wild-type and *TcITPK1*-SKO (SKO) gDNA digested with the PvuII restriction enzyme. The blot was hybridized with a biotin-labeled probe corresponding to 455 bp of *TcITPK1* 5’ UTR (nt −729 to −275), revealing a 2,700 bp band for PvuII-digested gDNA from WT cells and a 2,040 bp band for PvuII-digested gDNA from *TcITPK1*-SKO cells (arrows). (D) Growth of control (scrambled) and *TcITPK1*-SKO (*ITPK1*-SKO) epimastigotes in the LIT medium. (E) Percentage of metacyclic trypomastigotes in epimastigote cultures after incubation in the TAU 3AAG medium. Differentiation of epimastigotes to metacyclic trypomastigotes was quantified by staining with DAPI to distinguish the position of the kinetoplast by fluorescence microscopy. Values are expressed as means ± SD (*n* = 3) **P ≤ 0.01 by Student’s *t* test. (F) *TcITPK1*-SKO trypomastigote infection of Vero cells at 4 hours post-infection was significantly inhibited. Values are expressed as means ± SD (*n* = 3) *P ≤ 0.05 by Student’s *t* test. (G) The number of intracellular amastigotes per infected host cell observed 48 hours post-infection was also significantly reduced. Values are expressed as means ± SD (*n* = 3) ***P ≤ 0.001 by Student’s *t* test.

We then used a T7RNAP/Cas9 strategy to try to obtain *TcITPK1*-KO parasites. This cloning-free genome editing method involves co-transfecting a sgRNA template with a T7 promoter and donor DNA as PCR products (37). In addition, to generate a *null* mutant population using the T7RNAP/Cas9 system in a single round of electroporation, it is necessary to transfect cells with two donor DNAs, each one containing a different resistance marker (37). We employed a two-step approach: (i) first, we transfected T7RNAP/Cas9 epimastigotes with an sgRNA and one (PAC) or two (PAC and BSD) donor DNAs. (ii) If the knockout (KO) cell line was non-viable, a second transfection introduced the BSD cassette into a stable single-allele deletion mutant generated in the first round. We successfully generated a *TcITPK1* single-knockout (*TcITPK1*-SKO) cell line as both attempts (transfecting with two resistance cassettes simultaneously or using two sequential rounds of transfections) failed to produce *null* mutant cells for this gene. This result was confirmed through PCR (Fig. 4A and B) and Southern blot analysis (Fig. 4C; Fig. S5A). In addition, the growth rate of *TcITPK1*-SKO epimastigotes was not affected as compared with cells transfected with scrambled sgRNA (Fig. 4D). Metacyclogenesis, studied by incubating epimastigotes in triatome artificial urine, was stimulated in *TcITPK1*-SKO cells (Fig. 4E). We then infected Vero cells with tissue culture-derived trypomastigote stages and measured both the ability of trypomastigotes to infect host cells (Fig. 4F) and the replication of intracellular amastigotes (Fig. 4G), as described in Materials and Methods. Both trypomastigote invasion and amastigote replication were significantly affected by single knockout of *TcITPK1*, compared to control cells.

### Overexpression of *TcITPK1* in *T. cruzi* cells

**Fig 5:**
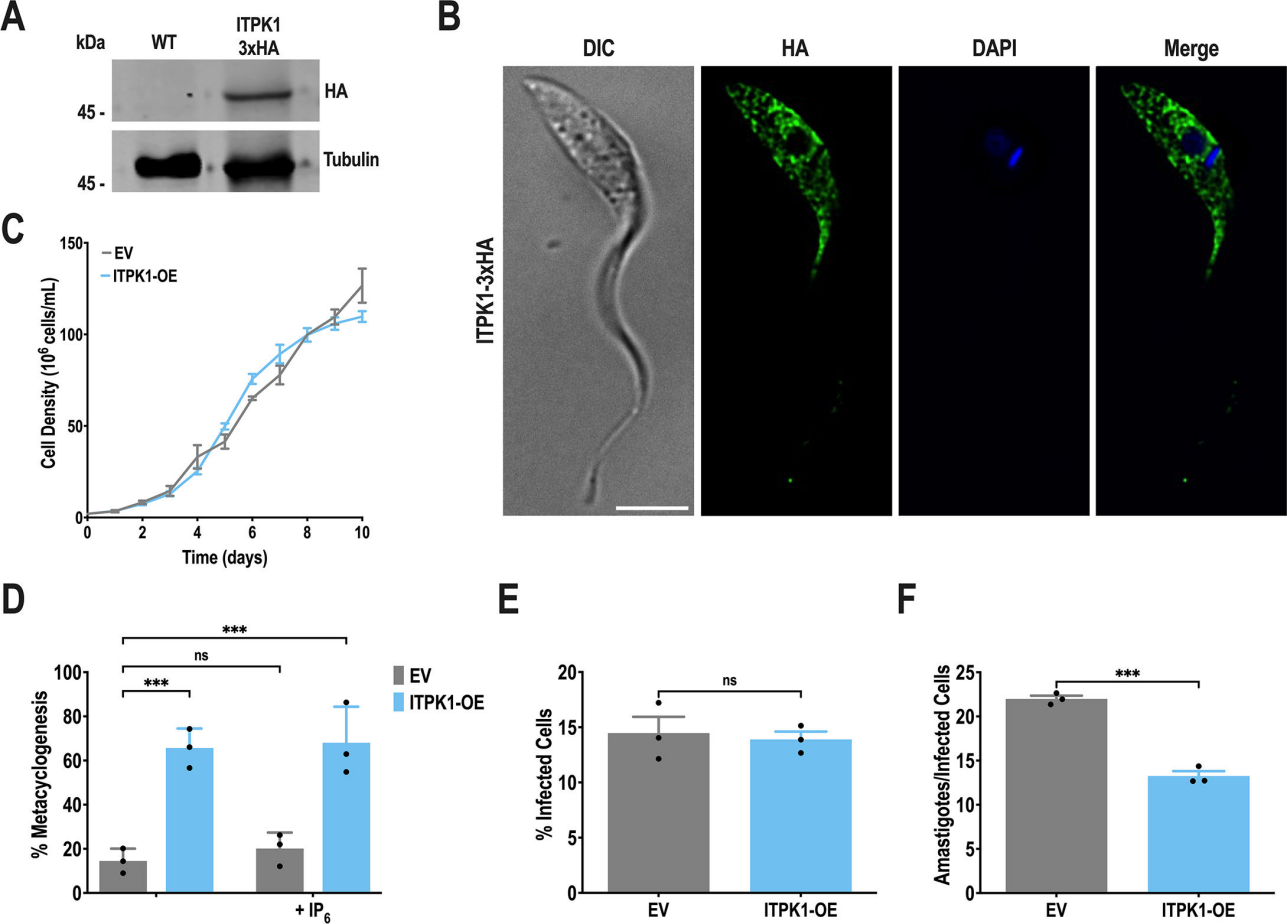
*TcITPK1* overexpression. (A) Western blot analysis of wild-type and TcITPK1-3×HA epimastigotes using monoclonal antibodies against the HA tag. The predicted protein molecular mass for TcITPK1-3×HA is 49 kDa. Molecular markers are on the left. Tubulin was used as a loading control. (B) Localization of TcITPK1-3×HA in epimastigotes using anti-HA antibodies. DIC, differential interference contrast. HA (green), TcITPK1-3×HA. The merge image shows TcITPK1-3×HA (green) and DAPI staining (blue). Scale bar = 5  µm. (C) Growth of control (EV, empty vector) and TcITPK1-3×HA (ITPK1-OE) epimastigotes in the LIT medium. (D) Percentage of metacyclic trypomastigotes in epimastigote cultures after incubation in the TAU 3AAG medium in the presence or absence of 150 µM IP_6_. Values are expressed as means ± SD (*n* = 3) ***P ≤ 0.001 by two-way ANOVA with Dunnett’s multiple-comparison test. (E) TcITPK1-3×HA trypomastigote infection of Vero cells at 4 hours post-infection was not significant. Values are expressed as means ± SD (*n* = 3) by Student’s *t* test. (F) The difference in the number of intracellular amastigotes per infected host cell observed 48 hours post-infection was significant. Values are expressed as means ± SD (*n* = 3) ***P ≤ 0.001 by Student’s *t* test.

To complete the functional study of TcITPK1, we also evaluated the effects of its upregulation in different stages of the *T. cruzi* life cycle by generating a mutant cell line (*TcITPK1*-OE) overexpressing the full-length (1,260 aa) C-terminal tagged protein (TcITPK1-3×HA). We evaluated the expression of TcITPK1-3×HA in a clonal population by Western blot analysis using anti-HA antibodies. The high-molecular weight signal detected in total protein extracts of *TcITPK1*-OE parasites corresponds to the predicted size of TcITPK1-3×HA (∼50 kDa) (Fig. 5A). Fluorescence microscopy images showed a localization pattern like that exhibited by the endogenous tagged protein (Fig. 5B). *TcITPK1*-OE epimastigotes had the same growth rate as control cells transfected with the pTREX-n empty vector (Fig. 5C). Notably, *TcITPK1*-OE exhibited a significantly increased capacity to differentiate *in vitro* into metacyclic trypomastigotes (Fig. 5D). Moreover, the ability of trypomastigotes to infect host cells was not affected (Fig. 5E), while the replication of intracellular amastigotes was significantly impaired by overexpression of *TcITPK1*, compared to control cells (Fig. 5F).

### Phospholipase C knockout in *T. cruzi* does not affect the synthesis of inositol polyphosphates

**Fig 6:**
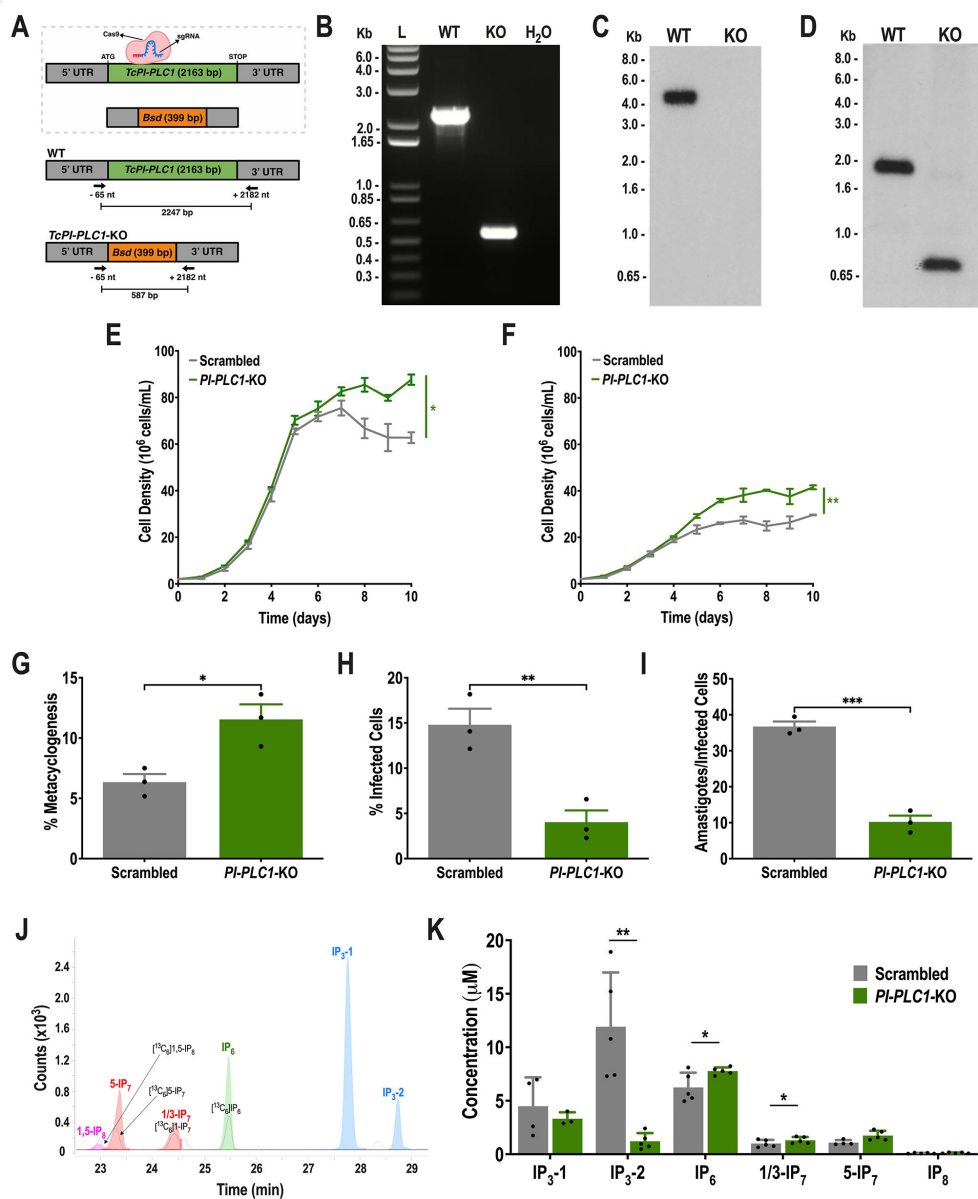
Knockout of *TcPI-PLC1*. (A) Schematic representation of the strategy used to generate a *TcPI-PLC1*-KO mutant by homologous recombination and primers (arrows) used to verify gene replacement by PCR. The intact locus generates a PCR product of 2,247 bp, while the disrupted locus generates a fragment of 587  bp. (B) PCR analysis showing that *TcPI-PLC1* was ablated at its genomic locus and replaced in genomic DNA of the KO cell line. Lanes: L, 1 kb plus ladder; WT, wild-type; KO, *TcPI-PLC1*-KO; H_2_O, PCR-negative control. (C) Southern blot analysis of wild-type and *TcPI-PLC1*-KO (KO) gDNA digested with the BamHI restriction enzyme. The blot was hybridized with a ^32^P-labeled probe corresponding to 439 bp of *TcPI-PLC1* (nt +1 to +439), revealing a 4,419-bp band only for BamHI-digested gDNA from WT cells. (D) Southern blot analysis of wild-type and *TcPI-PLC1*-KO (KO) gDNA digested with the PvuII restriction enzyme. The blot was hybridized with a ^32^P-labeled probe corresponding to 460 bp of *TcPI-PLC1* 5’ UTR (nt −503 to −1), revealing a 1,628-bp band for PvuII-digested gDNA from WT cells and a 715 bp band for PvuII-digested gDNA from *TcPI-PLC1*-KO cells. (E) Growth of control (scrambled) and *TcPI-PLC1*-KO (*PI-PLC1*-KO) epimastigotes in the LIT medium. Values are expressed as means ± SD (*n* = 3) *P ≤ 0.05 by Student’s *t* test. (F) Growth of control (scrambled) and *TcPI-PLC1*-KO (*PI-PLC1*-KO) epimastigotes in low-glucose LIT medium. Values are expressed as means ± SD (*n* = 3) **P ≤ 0.01 by Student’s *t* test. (G) Percentage of metacyclic trypomastigotes in epimastigote cultures after incubation in the TAU 3AAG medium. Values are expressed as means ± SD (*n* = 3) *P ≤ 0.01 by Student’s *t* test. (H) *TcPI-PLC1*-KO trypomastigote infection of Vero cells at 4 hours post-infection was significantly reduced. Values are expressed as means ± SD (*n* = 3) **P ≤ 0.01 by Student’s *t* test. (I) The number of intracellular amastigotes per infected host cell observed 48 hours post-infection was also significantly reduced. Values are expressed as means ± SD (*n* = 3) ***P ≤ 0.001 by Student’s *t* test. (J, K) Inositol phosphate extraction from scrambled and *TcPI-PLC1*-KO (*PI-PLC1*-KO) parasites, followed by CE-ESI-MS analysis, enables the identification of several important inositol phosphate and pyrophosphate isomers. (J) Separation of inositol phosphates by CE-ESI-MS from a *TcPI-PLC1*-KO sample. Black line: extracted ion electropherograms of [^13^C_6_] 1–5-IP_8_, 5-IP_7_, 1/3-IP7, and IP_6_ references; pink line: extracted electropherograms of IP_8_ in the sample; red line: extracted ion electropherograms of IP_7_ in the sample; green line: extracted ion electropherograms of IP_6_ in the sample; blue trace: extracted ion electropherograms of IP_3_ in the sample. (K) Concentration of inositol phosphates in scrambled and *TcPI-PLC1*-KO (*PI-PLC1*-KO) cells by CE-ESI-MS analysis shows that synthesis of IP_6_ and IP_7_ persists in *TcPI-PLC1*-KO epimastigotes. Values are expressed as means ± SD (*n* > 3) *P ≤ 0.01; **P ≤ 0.01 by Student’s *t* test.

To explore the role of phospholipase C in inositol polyphosphate synthesis, we designed a CRISPR/Cas9 strategy to generate KO mutants of this gene (Fig. 6A). After 5 weeks of selection with G418 and blasticidin, knockout of *TcPI-PLC1* was validated by PCR (Fig. 6B) and, after cloning by limiting dilution, by Southern blot analysis using probes comprising the 5′ end and the 5′ UTR (Fig. 6C and D; Fig. S5B).

Proliferation of epimastigotes was slightly stimulated in stationary-phase *TcPI-PLC1*-KO parasites either in the presence of regular (Fig. 6E) or low glucose levels (Fig. 6F). *TcPI-PLC1*-KO cells were able to differentiate to metacyclic trypomastigotes in a higher proportion than control cells (Fig. 6G). Furthermore, *TcPI-PLC1* knockout had a significant effect on trypomastigote infection of host cells (Fig. 6H) and amastigote replication (Fig. 6I).

We then investigated whether synthesis of inositol phosphates was affected in *TcPI-PLC1*-KO epimastigotes (Fig. 6J and K). Inositol phosphates were extracted from *TcPI-PLC1*-KO cells and control cells transfected with scrambled sgRNA using titanium beads, following the previously described method (38). The extracted inositol polyphosphates were then analyzed by capillary electrophoresis-electrospray ionization-mass spectrometry (CE-ESI-MS) (39, 40), using ^13^C-labeled internal references (41). Fig. 6K shows the detection of 5-IP_7_ in epimastigote extracts of *T. cruzi*. Interestingly, an IP_7_ not previously identified (pyrophosphorylated in C-1 or C-3, as they are enantiomers and thus indistinguishable by CE-MS) in *T. cruzi* was detected (Fig. 6J). Fig. 6K shows that the levels of IP_6_, 1/3-IP_7_, and 5-IP_7_ were not decreased in the *TcPI-PLC1*-KO cells; instead, levels of IP_6_ and 1/3-IP_7_ were slightly increased, while levels of IP_3_-2 did decrease. In conclusion, TcPI-PLC1 does not appear relevant for the synthesis of inositol polyphosphates in *T. cruzi* epimastigotes, although it is important for IP_3_-2 formation.

**Fig 7:**
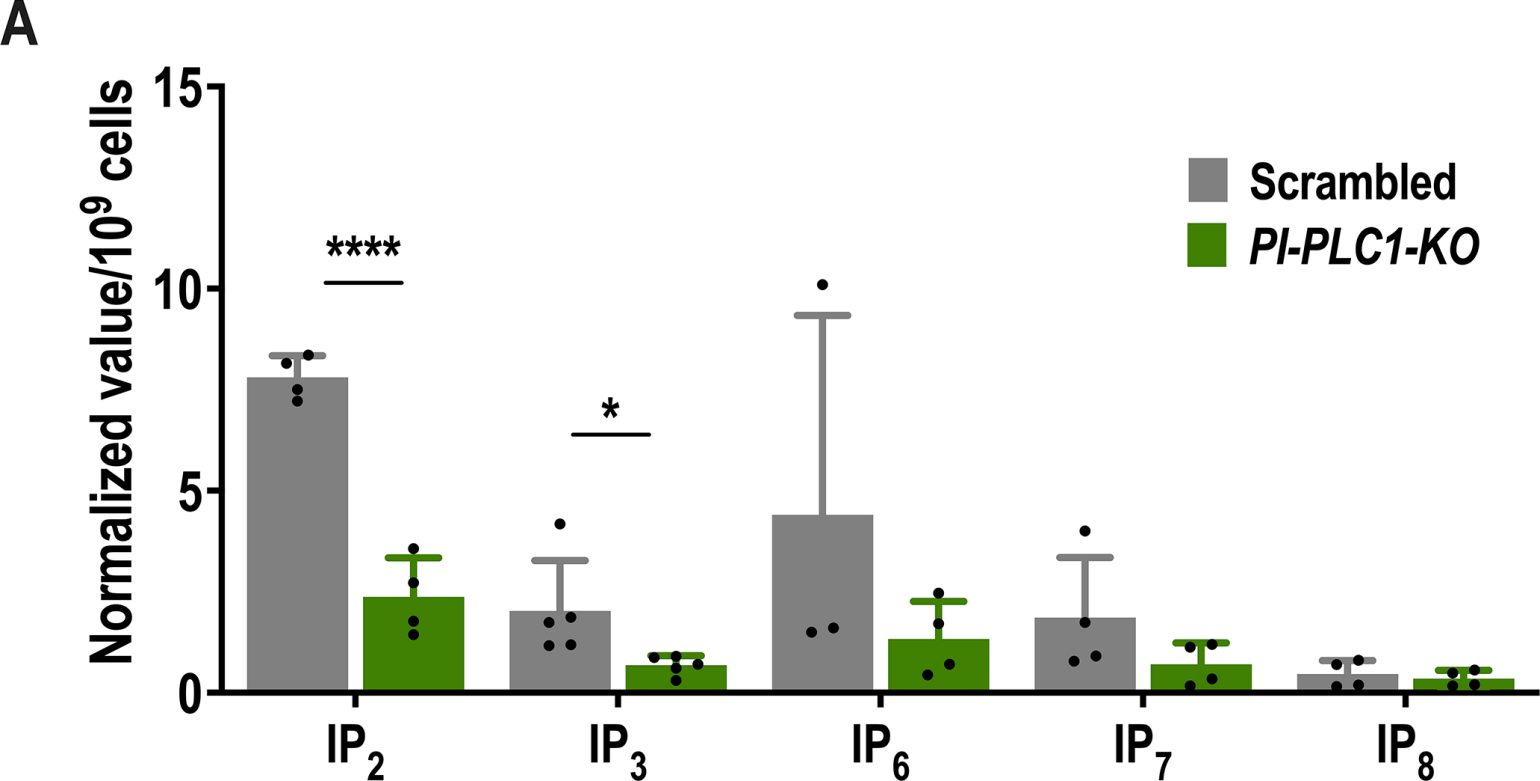
Glucose labeling for inositol phosphate detection. (A) Metabolic labeling was performed by cultivating *T. cruzi* epimastigotes at an initial density of 2 × 10⁶ cells/mL in low-glucose LIT medium (no added glucose) for 48 hours. After this period, the parasites were harvested by centrifugation and resuspended in fresh LIT medium containing either 10 mM ¹³C-labeled D-glucose or 10 mM unlabeled D-glucose. The cultures were incubated for an additional 48 hours. Subsequently, the cells were harvested by centrifugation for inositol phosphate extraction and LC-MS analysis. Values are expressed as means ± SD (*n* > 3) *P ≤ 0.05; ****P ≤ 0.0001 by Student’s *t* test.

Having seen that a PLC-independent pathway is operative in *T. cruzi*, we devised a set of experiments aimed at defining which alternative substrates could feed the IP pool. To investigate whether 3-IP_1_ produced from glucose 6-phosphate by the inositol 3-phosphate synthase could also be used *in vivo* as a substrate of ITPK1 to generate inositol polyphosphates, we grew wild-type and *TcPI-PLC1*-KO *T. cruzi* epimastigotes with 1-^13^C-D-glucose and measured the formation of inositol phosphates. These KO cells were able to synthesize IP_6-8_ from glucose, as detected by LC-MS (19) of epimastigote extracts (Fig. 7), demonstrating the presence of the alternative pathway for the synthesis of IPs from G6P. Interestingly, while labeling of IP_2_ and IP_3_ decreased significantly in the *TcPI-PLC1*-KO mutants, no significant differences were detected in the labeling of IP_6-8_, suggesting that the lipid-dependent pathway is important for the formation of IP_3_ but not for the formation of IP_6-8_ under these conditions (Fig. 7).

### *TcISC1* knockout cells produce less IP_6_ and are unable to infect tissue culture cells

**Fig 8:**
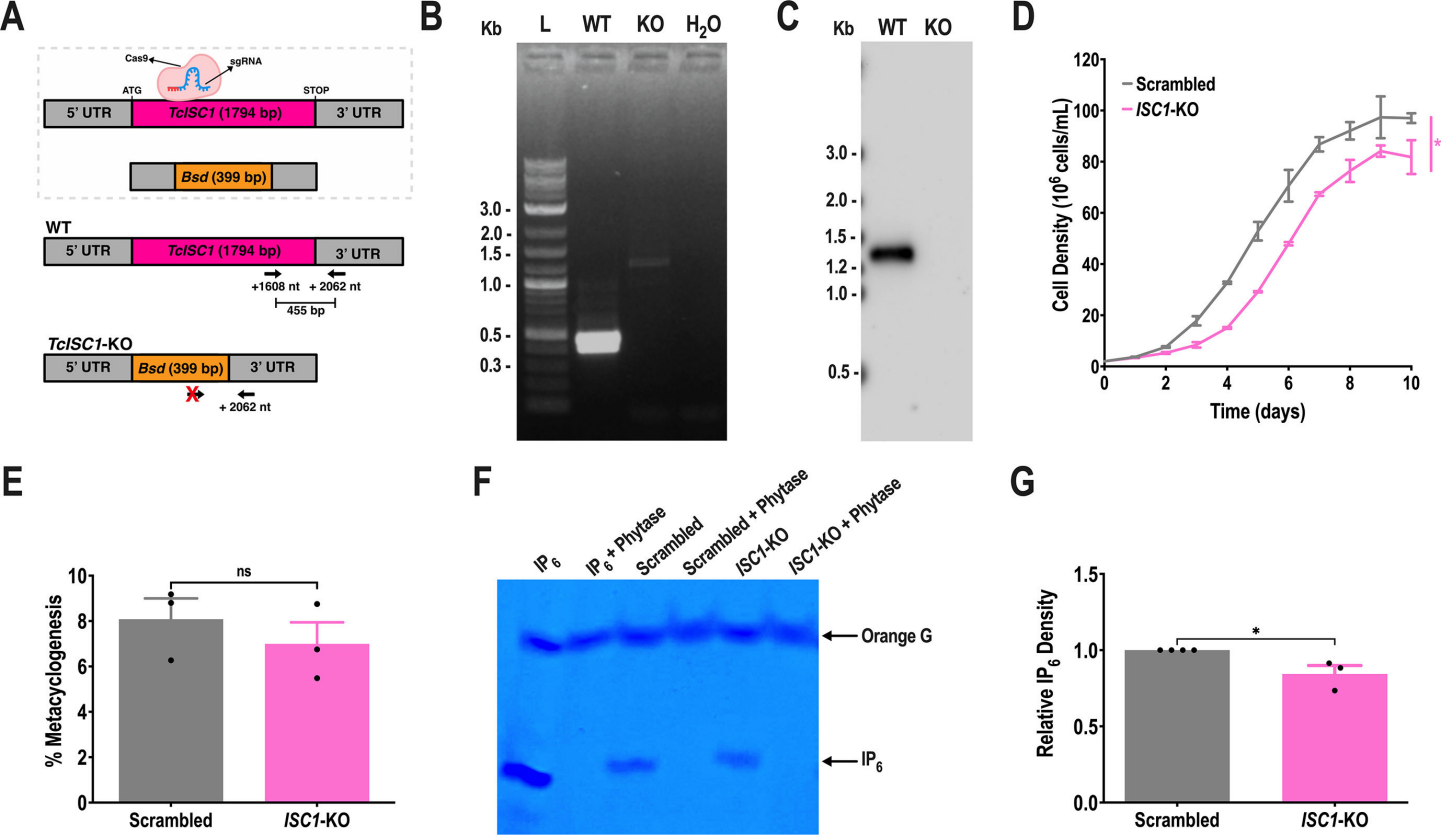
Knockout of *TcISC1*. (A) Schematic representation of the strategy used to generate a *TcISC1*-KO mutant by homologous recombination and primers (arrows) used to verify gene replacement by PCR. The intact locus generates a PCR product of 455 bp, while the disrupted locus does not generate a fragment. (B) PCR analysis showing that *TcISC1* was ablated at its genomic locus and replaced in genomic DNA of the KO cell line. Lanes: L, 1 kb plus ladder; WT, wild-type; KO, *TcISC1*-KO; H_2_O, PCR-negative control. (C) Southern blot analysis of wild-type and *TcISC1*-KO (KO) gDNA digested with the PvuII restriction enzyme. The blot was hybridized with a biotin-labeled probe corresponding to 435 bp of *TcISC1* (nt +694 to +1,128), revealing a 1,295-bp band only for PvuII-digested gDNA from WT cells. (D) Growth of control (scrambled) and *TcISC1*-KO (*ISC1*-KO) epimastigotes in the LIT medium. Values are expressed as means ± SD (*n* = 3) *P ≤ 0.05 by Student’s *t* test. (E) Percentage of metacyclic trypomastigotes in epimastigote cultures after incubation in the TAU 3AAG medium. Values are expressed as means ± SD (*n* = 3) by Student’s *t* test. (F) PAGE analysis of IP_6_ and IP_6_ extracts from scrambled and *TcISC1*-KO epimastigotes. Half of the samples were treated with phytase (0.1 mg/mL, pH 5.0, at 37°C for 1 hours) to confirm that the bands correspond to IP_6_. (G) Densitometry of toluidine-stained IP_6_ from scrambled and *TcISC1*-KO. Values are expressed as means ± SD (*n* = 3) *P ≤ 0.05 by Student’s *t* test.

To investigate whether the expression of *TcISC1* was important to generate 1-IP_1_ from inositol phosphoceramide (IPC) as a source of inositol polyphosphates, we utilized a T7RNAP/Cas9 strategy to generate KO mutants of this gene (Fig. 8A). The KO was validated by PCR (Fig. 8B) and Southern blot analysis (Fig. 8C; Fig. S5C). Proliferation of *TcISC1*-KO epimastigotes was significantly affected (Fig. 8D) but metacyclogenesis was not (Fig. 8E). To investigate the importance of TcISC1 to produce IP_6_, we extracted IPs from large amounts of cells and tested extracts by 35% polyacrylamide gel electrophoresis. A band that runs like the IP_6_ standard and that disappears after treatment of the extracts with phytase was detected. The densitometric analysis revealed that the band was significantly decreased in *TcISC1*-KO cells, revealing that TcISC1 participates in the synthesis of IP_6_ through the conversion of IPC into 1-IP_1_ (Fig. 8F and G). Several attempts (*n* = 3) to infect tissue culture cells with *TcISC1*-KO metacyclic forms failed and did not result in recovery of cell-derived trypomastigotes, suggesting that *TcISC1* is important for infectivity.

**Fig 9:**
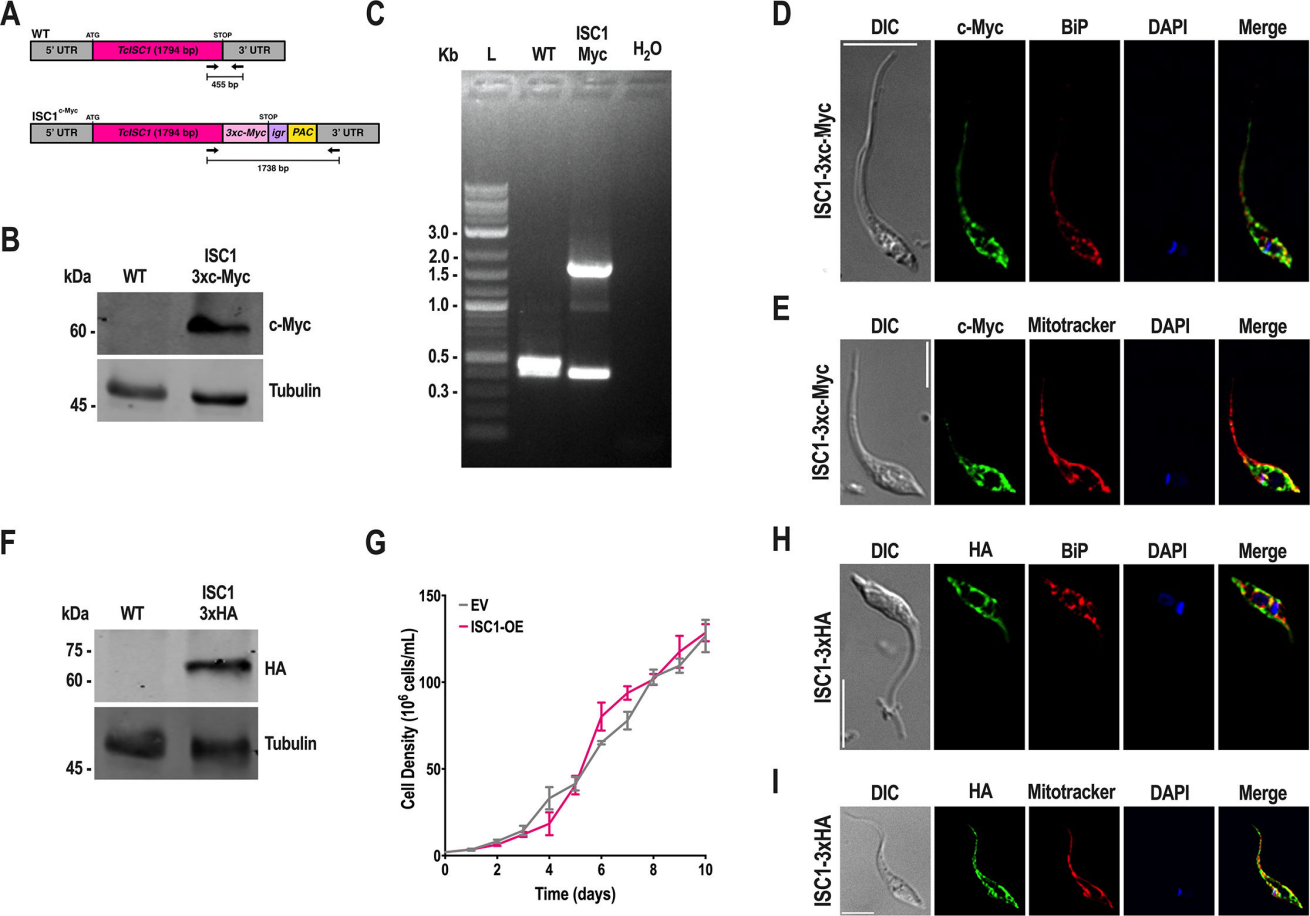
CRISPR/Cas9 endogenous C-terminal tagging and overexpression of *TcISC1*. (A) Schematic representation of the wild-type and endogenous tagged *TcISC1* gene. Epimastigotes were endogenously tagged with a 3×c-Myc tag using CRISPR/Cas9 genome editing. (B) PCR analysis for validation of *TcISC1* tagging showing expected bands for control cell lines (WT, predicted size, 455 bp) and *TcISC1*-3×c-Myc cell line (ISC1 Myc, predicted size, 1,738 bp). (C) Western blot analysis of wild-type and TcISC1-3×c-Myc epimastigotes using monoclonal antibodies against c-Myc tag. The predicted protein molecular mass for TcISC1-3×c-Myc was 72 kDa. Molecular markers are on the left. Tubulin was used as a loading control. (D) Localization of endogenously tagged TcISC1-3×c-Myc in epimastigotes using anti-c-Myc antibodies. DIC, differential interference contrast. c-Myc (green), TcISC1-3×c-Myc. BiP (red), endoplasmic reticulum marker. The merge image shows that TcISC1-3×c-Myc (green) colocalizes with BiP (red); Pearson’s correlation coefficient (PCC) = 0.74. Scale bar = 5  µm. (E) Localization of endogenously tagged TcISC1-3×c-Myc in epimastigotes using anti-c-Myc antibodies. DIC, differential interference contrast. c-Myc (green), TcISC1-3×c-Myc. Mitotracker (red), mitochondrial marker. The merge image shows that TcISC1-3×c-Myc (green) colocalizes with Mitotracker (red); PCC = 0.60. Scale bar = 5  µm. (F) Western blot analysis of wild-type and TcISC1-3×HA epimastigotes using monoclonal antibodies against the HA tag. The predicted protein molecular mass for TcISC1-3×HA is 69 kDa. Molecular markers are on the left. Tubulin was used as a loading control. (G) Growth of control (EV, empty vector) and TcISC1-3×HA (ISC1-OE) epimastigotes in the LIT medium. (H) Localization of TcISC1-3×HA in epimastigotes using anti-HA antibodies. DIC, differential interference contrast. HA (green), TcISC1-3×HA. BiP (red), endoplasmic reticulum marker. The merge image shows that TcISC1-3×HA (green) colocalizes with BiP (red); PCC = 0.67. Scale bar = 5  µm. (I) Localization of TcISC1-3×HA in epimastigotes using anti-HA antibodies. DIC, differential interference contrast. HA (green), TcISC1-3×HA. Mitotracker (red), mitochondrial marker. The merge image shows that TcISC1-3×HA (green) colocalizes with Mitotracker (red); PCC = 0.76. Scale bar = 5  µm.

C-terminal tagging of TcISC1 (Fig. 9A) was validated by Western blot analysis (Fig. 9B) and PCR (Fig. 9C), and the enzyme was found to co-localize with the endoplasmic reticulum marker BiP (Fig. 9D) and the mitochondrial marker MitoTracker (Fig. 9E). Overexpression of *TcISC1* (Fig. 9F) did not affect epimastigote growth (Fig. 9G) and confirmed the dual localization in the ER (Fig. 9H) and mitochondria (Fig. 9I).

## DISCUSSION

The main finding of this work is that in *T. cruzi,* the pathway for the formation of inositol phosphates using the TcPI-PLC1 is mainly involved in IP_3_ formation, which is crucial for Ca^2+^ signaling, trypomastigote invasion of host cells, and amastigote replication. Meanwhile, the PI-PLC1-independent, or cytosolic, pathway is involved in the formation of inositol polyphosphates from endogenous sources via glucose 6-phosphate or inositolphosphoceramide, leading to inositol monophosphate production. This pathway is essential for parasite survival.

The hypothetical protein TcYC6_0083580 was identified as an inositol tetrakisphosphate kinase 1 (ITPK1). We used a bioinformatic toolset to establish the evolutionary relationship and structural conservation of TcITPK1. Initial results of multiple alignment of protein sequences demonstrated that TcITPK1 shares two out of three highly conserved residues and three out of four less conserved residues required for inositol phosphate- and ATP-binding of the protein.

The phylogenetic study demonstrated that ITPK1 is present in kinetoplastids and other higher-order eukaryotes and that most critical residues are conserved. This finding was also noted in a recent review on the inositol phosphate pathway in higher-order eukaryotes (42). *T. cruzi* is found in the Discoba supergroup of eukaryotes, whereas *Homo sapiens* is a member of the Opisthokonta supergroup (43). While both organisms are in different eukaryotic supergroups, the evidence suggests that their last eukaryotic common ancestor had this lipid-independent, cytosolic pathway to synthesize inositol polyphosphates from glucose 6-phosphate without the need for PLC. The absence of this pathway in apicomplexans appears as a recent acquisition of that clade and does not reflect the overall evolution of this pathway.

Immunofluorescent assays of CRISPR/Cas9 endogenously tagged *TcITPK1* demonstrated that TcITPK1 localizes to the cytosol. The failed attempts to generate a CRISPR/Cas9-mediated *TcITPK1* knockout and knockdown suggest that *TcITPK1* may be an essential gene in *T. cruzi* epimastigotes and that the lipid-independent pathway plays an important role in parasite survival. In this regard, the INO1 has been shown to be essential for their survival (44), while we found that *TcISC1*-KO cells are unable to infect mammalian cells.

TcPI-PLC1 is lipid-modified at its N-terminus and plays an essential role in cell signaling (45–48). We were able to knockout *TcPI-PLC1* in epimastigotes despite previous unsuccessful attempts that suggested its essentiality (47). However, the proliferation of *TcPI-PLC1*-KO epimastigotes was affected, while trypomastigote host cell invasion and intracellular amastigote replication were significantly reduced. The effect on amastigote replication could be related to the inhibition of trypomastigote to amastigote differentiation observed when *TcPI-PLC1* expression was downregulated by antisense oligonucleotides (47).

*TcPI-PLC1* genetic knockout in *T. cruzi* epimastigotes does not stop the formation of higher-order inositol phosphates, demonstrating the existence of an alternative synthesis pathway. *S. cerevisiae* genetic screens were employed to determine its capabilities to phosphorylate inositol monophosphate and other inositol phosphate species. SAX-HPLC experiments demonstrated that complementation of *S. cerevisiae plc1*Δ with *TcITPK1* rescues the synthesis of IP_6_. This result is also in agreement with previous results on HsITPK1 (22) and the positive control. There are three highly conserved residues that are important for IP- and ATP-binding—H167, K199, and R212—in HsITPK1. TcITPK1 only shares two of these conserved residues, and therefore this may explain the differences in the overall rescue of IP_6_ synthesis. However, attempts to knockout the lipid-independent inositol phosphate synthesis pathway in *TcITPK1*-KO experiments resulted in the death of parasite cultures, so this lesser rescue was unexpected. In contrast, *S. cerevisiae* growth assays demonstrate TcITPK1 was able to rescue the growth of *S. cerevisiae plc1*Δ. This impact may be due to the formation of inositol phosphate species, an important molecule to a variety of yeast cellular processes including regulation of major glycolytic transcription factor GCR1, pseudohyphal growth, and ATP concentration (49, 50). The *S. cerevisiae* phosphoinositide-specific phospholipase C gene (*PLC1*) is a homolog to *TcPI-PLC1* and other delta class PLC enzymes, and the knockdown of *plc* causes organisms to over accumulate PIP_2_ and fail to synthesize inositol phosphate (22). The lagging growth phenotype in *myo*-inositol-deficient media further demonstrates the importance of inositol phosphate species synthesis as these samples took longer to reach the stationary phase. However, complementation with either eukaryotic ITPK1 enzymes—be it TcITPK1 or HsITPK1—allowed for the rescue of this growth deficiency.

As it has been proposed before (22), the occurrence of two independent pathways for inositol phosphate metabolism, one associated with the formation of the Ca^2+^ signaling agent IP_3_ and the other associated with the production of inositol pyrophosphates, suggests different compartmentalization. In this regard, pools of independently cycling inositol phosphates have been reported in human cells (51). This is supported by results in *T. brucei*, where uptake of [^3^H]inositol did not result in detection of IP_6_, while IP_6_ was easily detected by PAGE analyses suggesting an endogenous origin (18). In addition, it has been demonstrated that in *T. brucei,* the formation of phosphoinositides and IP_3_ depends on exogenous inositol uptake, while synthesis of GPI anchors requires endogenous synthesis of inositol. As we described in Fig. 1, endogenous synthesis of inositol phosphates could occur by the conversion of G6P into 3-IP_1,_ catalyzed by INO1, or by the conversion of IPC into 1-IP_1,_ catalyzed by ISC1. In this work, we demonstrate that TcITPK1 can use both 1-IP_1_ generated from IPC in yeast or *T. cruzi* and 3-IP_1_ generated from G6P in *T. cruzi*. The production of 1-IP_1_ from IPC could be important in *T. cruzi* because IPC is abundant in characteristic GPI anchors of *T. cruzi,* like those in the *trans*-sialidase of trypomastigotes (52), and the surface protein Ssp4 of amastigotes (53). IPC is also present in the anchor of mucins of metacyclic trypomastigotes (54). A free glycoinositol phospholipid (GIPL) originally named lipopeptide phosphoglycan (LPPG) is the major glycoconjugate of *T. cruzi* epimastigotes and also has IPC (55).

Another potential source of IPC is by salvage of lipids from the host cells and remodeling in the mammalian stages, as occurs in *Leishmania* (10, 11). Knockout of *L. mexicana* IPC synthase affects the synthesis of IP_2_ and IP_6_ (56), which agrees with the present study’s findings. Disruption of *T. cruzi* IPC synthase has been shown to affect metacyclogenesis, intracellular amastigote proliferation, and differentiation of amastigotes into tissue culture-derived trypomastigotes, preventing the establishment of infection *in vivo* in immune-deficient mice (57). In agreement with that report, we found that *TcISC1*-KO parasites were unable to infect host cells. This effect could be related to the reported hypersensitivity to acidic stress of knockout mutants of *Isc1* in *Cryptococcus neoformans* (58) and *Leishmania major (59)*, which prevents their proliferation in the macrophage phagolysosomes. In this regard, *T. cruzi* also occupies an acidic phagolysosome upon entry into the host cells (60), which is needed to differentiate the infective trypomastigotes into replicating amastigotes. As occurs with *S. cerevisiae* ISC1 (61, 62), TcISC1 has a dual localization in the ER and mitochondria.

In summary, while the TcPLC1 pathway is important for generating IP_3_, which is needed for Ca^2+^ signaling, the TcPLC1-independent, or cytosolic, pathway is involved in the generation of inositol polyphosphates. The essential role of this pathway in the infection of host cells by *T. cruzi* suggests that the enzymes involved could serve as potential drug targets, indicating the possibility of developing inhibitors.

## MATERIALS AND METHODS

### Clustal omega analysis

Ten homologous ITPK1 amino acid sequences of interest were identified from *Lokiarchaeum candidatus*, *Entamoeba histolytica, Oryza sativa, Zea mays, Monosiga brevicollis, Dictyostelium discoideum, Danio rerio, Gallus gallus, Homo sapiens,* and *Mus musculus* from a previous publication (22). A potential TcITPK1 homolog was identified in TriTrypDB as TcYC6_0083580 (GenBank KAF8297109.1) (63). A FASTA file containing all eleven homologous amino acid sequences was uploaded to Clustal Omega Multiple Sequence Alignment, and default multiple sequence alignment parameters were used (64).

### Constructing an ITPK1 phylogenetic tree

The initial amino acid sequence for *T. cruzi* inositol tetrakisphosphate 1-kinase (TcITPK1) was obtained from TriTrypDB. TriTrypDB and OrthoMCL search analyses were performed using the full-length protein sequence of the *T. cruzi* ITPK1 protein (TcYC6_0083580) and orthology group number (OG6_147480) (63, 65). In addition, five selected ITPK1 predicted orthologs were added to the compilation of identified orthologs for a total of 27 unique sequences. The bootstrap consensus tree inferred from 1,000 replicates was taken to represent the evolutionary history of the taxa analyzed. Branches corresponding to partitions reproduced in less than 50% bootstrap replicates are collapsed. The percentage of replicate trees in which the associated taxa clustered together in the bootstrap test (1,000 replicates) were shown next to the branches. The evolutionary distances were computed using the JTT matrix-based method and are expressed in the units of the number of amino acid substitutions per site. The rate variation among sites was modeled with a gamma distribution (shape parameter = 4). All ambiguous positions were removed for each sequence pair (pairwise deletion option). There was a total of 773 positions in the final data set. Evolutionary analyses were conducted in MEGA11.

### AlphaFold-2.1.1 prediction and modeling of TcITPK1 and HsITPK1

AlphaFold Version 2.1.1 was installed on the Sapelo2 cluster by the Georgia Advanced Computer Center (GACRC) of the University of Georgia using EasyBuild following the steps in the dockerfile available at (25). The run_alphafold.sh bash script was obtained from https://github.com/kalininalab/alphafold_non_docker, and related documentation is available at that URL. The run_alphafold.sh bash script was edited to run the FASTA file containing the amino acid sequence for either TcITPK1 or HsITPK1 using the monomer_casp14 modeling parameters.

### Protein structural comparison analysis

Pairwise structure alignment is a tool on the RCSB Protein Data Bank website that allows for the comparison of two or more protein structures (28–34). As a built-in control for AlphaFold-2.1.1 prediction accuracy, the best model for HsITPK1 predicted by the AlphaFold-2.1.1 Monomer_Casp14 algorithm was compared to the X-ray diffraction-resolved HsITPK1 (2ODT) structure using the jFATCAT (flexible) parameter (35). Pairwise structural alignment outputs a table describing the RMSD, TM-Score, Score, SI%, SS%, and overall length of the structures selected/uploaded for superposition. Structural comparison was repeated at FATCAT using the FATCAT (flexible) to find the significance of structural comparisons (33). This protein structural comparison analysis pipeline was repeated for the best AlphaFold-2.1.1-predicted model for TcITPK1 and X-ray diffraction-resolved HsITPK1 (2ODT) structure.

### Chemical and reagents

Platinum Taq DNA Polymerase High-Fidelity, BCA protein assay kit, Alexa-conjugated secondary antibodies, MitoTracker deep red FM, Chemiluminescent Nucleic Acid Detection Module, and BioPrime DNA Labeling System kit were obtained from Thermo Fisher Scientific (Waltham, MA). The BLUelf prestained protein ladder was obtained from FroggaBio (Wheatfield, NY). The anti-c-Myc monoclonal antibody (clone 9E10) was from Santa Cruz Biotechnology (Dallas, TX). Puromycin was from Acros Organics (Fair Lawn, NJ). Blasticidin S HCl was purchased from Life Technologies (Grand Island, NY). Wizard plus SV miniprep Purification System, Wizard SV Gel and PCR Clean-up System, GoTaq DNA polymerase, and T4 DNA ligase were from Promega (Madison, WI). Antarctic phosphatase, restriction enzymes, and Q5 High-Fidelity DNA Polymerase were from New England Biolabs (Ipswich, MA). Fluoromount-G was from SouthernBiotech (Birmingham, AL). The primers were purchased from Integrated DNA Technologies. The Nitrocellulose Membranes were from Bio-Rad (Hercules, CA). G418 sulfate was from KSE Scientific (Durham, NC). The anti-HA mouse monoclonal antibody was from BioLegend (San Diego, CA). Anti-tubulin monoclonal antibody, anti-glutathione-S-transferase rabbit polyclonal antibody, mammalian cell protease inhibitor mixture (Sigma P8340), other protease inhibitors, Benzonase Nuclease, and all other reagents of analytical grade were from Sigma (St. Louis, MO). The rabbit polyclonal antibody against TbBiP (66) was provided by Dr. Jay Bangs (University at Buffalo, NY).

### Culture methods

*T. cruzi* (Y strain) epimastigotes were maintained at 28°C in the LIT medium (67), supplemented with 10% newborn calf serum (NCS), penicillin (100 U mL^−1^), and streptomycin (100 µg mL^−1^). Mutant cell lines were maintained in the medium containing 250  µg/mL G418, 10  µg/mL blasticidin, or 5  µg/mL puromycin. The growth rate of epimastigotes was determined by counting cells every 24 hours using a Coulter Counter (Beckman Coulter). Tissue culture cell-derived trypomastigotes were obtained from Vero cells infected with metacyclic trypomastigotes as described below. *T. cruzi* trypomastigote forms were collected from the culture medium of infected host cells, using a modification of the method of Schmatz and Murray (68), as described previously (69). Vero cells were grown in RPMI supplemented with 10% fetal bovine serum (FBS) and maintained at 37°C with 5% CO_2_.

### *TcITPK1* and *TcISC1* overexpression

*TcITPK1* and *TcISC1* open reading frames (ORF) (1,260 and 1,794 nt, respectively) were PCR-amplified using *T. cruzi* Y strain gDNA as the template (primers 31, 32, 33, and 34; Table S1) and cloned into the pTREX-n/3×HA vector by restriction sites XbaI/XhoI. Gene cloning was confirmed by PCR and sequencing, and constructs were subsequently used to transfect *T. cruzi* epimastigotes. *TcITPK1* and *TcISC1* overexpression was confirmed by Western blot analysis using anti-HA antibodies.

### CRISPR/Cas9 endogenous C-terminal tagging.

Targeted sgRNAs for endogenous C-terminal tagging (3′-end) were amplified in a one-step PCR using a forward oligonucleotide primer (Table S1, primer 10 or 42), universal reverse primer (Table S1, primer 45), and pUC_sgRNA plasmid as a template. This PCR step was followed by PCR purification. The specific sgRNA was cloned into the Cas9/pTREX-n vector (Addgene plasmid #68708) (36), and alignment was verified by Sanger sequencing (Table S1, primer 46). Donor DNA was synthesized for endogenous C-terminal tagging in a one-step PCR using the pMOTag23M DNA vector as the DNA template and long ultramers (Table S1, primers 11–12 and 43–44). After visualization of donor DNA on an agarose gel, DNA was purified by phenol/chloroform/isoamyl alcohol (25:24:1) extraction and quantified by NanoDrop spectrophotometry. Epimastigotes were co-transfected with sgRNA/Cas9/pTREX-n and DNA donor and then cultured for 5 weeks with G418 and puromycin for selection of double-resistant parasites. Endogenous gene tagging was verified by PCR from gDNA using specific primer sets (Table S1, primers 13–14 and 38–39) and by Western blot analysis.

### Knockout of *TcPI-PLC1*

Chimera single-guide RNA (sgRNA) sequences to target the *TcPI-PLC1* gene (TcYC6_0042910; AB022677.1) were PCR-amplified (Table S1, primers 1 and 47) from plasmid pUC_sgRNA, as previously described (36). Selection of the protospacer was performed using EuPaGDT (Eukaryotic Pathogen CRISPR guide RNA Design Tool, http://grna.ctegd.uga.edu/). The protospacer sequence was included in the forward primer, while using a common reverse primer for sgRNA amplification. The sgRNA orientation was verified by PCR using the specific *TcPI-PLC1*-sgRNA forward primer and the HX1 reverse primer (Table S1, primers 1 and 46) (36). Positive clones that generate a 190-bp PCR fragment were also sequenced. A scrambled sgRNA (scrambled-sgRNA/Cas9/pTREX-n) was used as control. A DNA donor cassette designed to promote homologous directed repair and replacement of *TcPI-PLC1* ORF was obtained by PCR using a set of long primers (ultramers) containing 120 nucleotides, from which 100 nucleotides correspond to the first 100 nt (forward ultramer) and the last 100 nt (reverse ultramer) of *TcPI-PLC1* ORF, and 20 nt annealing on the blasticidin gene (Table S1, primers 2 and 3). The *TcPI-PLC1-*sgRNA/pTREX-p construct and linear *blasticidin* cassette were used to transfect epimastigotes. After 5 weeks of selection with 250 µg/mL G418 and 10 µg/mL blasticidin, *TcPI-PLC1* gene replacement was verified by PCR using primers 8 and 9 (Table S1).

### Knockout of *TcITPK1*

The knockout strategy using the T7RNAP/Cas9 was designed and performed as previously described (37). Chimera single-guide RNA (sgRNA) sequences to target the *TcITPK1* gene (TryTripDB identifier [ID] TcYC6_0083580) were PCR-amplified (Table S1, primers 15 and 16). Selection of the protospacer was performed using EuPaGDT (Eukaryotic Pathogen CRISPR guide RNA Design Tool, http://grna.ctegd.uga.edu/). The protospacer sequence and the T7 polymerase-binding site were included in the forward primer, while using a common reverse primer for sgRNA amplification. A DNA donor cassette designed to promote homologous directed repair and replacement of *TcITPK1* ORF was obtained by PCR using a set of primers (Table S1, primers 17 and 18). The forward primer contains a 40-nt 5′UTR-containing homologous region (HR) plus 20 nucleotides of the plasmid backbone including the start codon (5′-GCCGCGGGAATTCGATTATG-3′). The reverse primers consist of 37 nucleotides of the 3′UTR-containing HR followed by 23 nucleotides of the plasmid backbone and the four last nucleotides of the antibiotic resistance genes, including the stop codon (5′-CGCGAATTCACTAGTGATTTCAC-3′). To amplify the donor DNA cassettes, the plasmids p*BSD* or p*PAC* were used as templates. We co-transfected *T. cruzi* T7RNAP/Cas9 epimastigotes with the sgRNA template and one (puromycin) or two (puromycin and blasticidin) donor DNAs. Transfected parasites were cultured for 4 weeks in the presence of G418 and puromycin, or G418, puromycin, and blasticidin for selection of single- or double-KO parasites, respectively. Gene disruption was verified using gDNA from mutant parasites by PCR using primers 19 and 20 (Table S1).

### Knockout of *TcISC1*

The knockout strategy using the T7RNAP/Cas9 was designed and performed as previously described (37). Chimera single-guide RNA (sgRNA) sequences to target the *TcISC1* gene (TcYC6_0066130; KAF8297109.1) were PCR-amplified (Table S1, primers 15 and 35). Selection of the protospacer was performed using EuPaGDT (Eukaryotic Pathogen CRISPR guide RNA Design Tool, http://grna.ctegd.uga.edu/). The protospacer sequence and the T7 polymerase-binding site were included in the forward primer, while using a common reverse primer for sgRNA amplification. A DNA donor cassette designed to promote homologous directed repair and replacement of *TcISC1* ORF was obtained by PCR using a set of primers (Table S1, primers 36 and 37). The forward primer contains a 40-nt 5′UTR-containing homologous region (HR) plus 20 nucleotides of the plasmid backbone including the start codon (5′-GCCGCGGGAATTCGATTATG-3′). The reverse primers consist of 37 nucleotides of the 3′UTR-containing HR followed by 23 nucleotides of the plasmid backbone and the four last nucleotides of the antibiotic resistance genes, including the stop codon (5′-CGCGAATTCACTAGTGATTTCAC-3′). To amplify the donor DNA cassettes, the plasmids p*BSD* or p*PAC* were used as templates. We co-transfected *T. cruzi* T7RNAP/Cas9 epimastigotes with the sgRNA template and one (puromycin) or two (puromycin and blasticidin) donor DNAs. Transfected parasites were cultured for 4 weeks in the presence of G418 and puromycin, or G418, puromycin, and blasticidin, for selection of single- or double-KO parasites, respectively. Gene disruption was verified using gDNA from mutant parasites by PCR using primers 38 and 39 (Table S1).

### Cell transfection

Transfections were performed as previously described (70). Briefly, *T. cruzi* Y strain epimastigotes (4 × 10^7^ cells) were washed with phosphate-buffered saline (PBS), pH 7.4, at room temperature (RT) and transfected in ice-cold CytoMix (120 mM KCl, 0.15 mM CaCl_2_, 10 mM K_2_HPO_4_, 25 mM HEPES, 2 mM EDTA, 5 mM MgCl_2_, pH 7.6) containing 25 µg of each plasmid construct in 4-mm electroporation cuvettes with three pulses (1,500 V, 25 µF) delivered by a Gene Pulser Xcell Electroporation System (Bio-Rad). Stable cell lines were established and maintained under drug selection with appropriate antibiotic(s) (250 µg/mL G418, 10 µg/mL blasticidin, and/or 5 µg/mL puromycin). Transfectant epimastigotes were cultured in the LIT medium supplemented with 20% heat-inactivated NCS until stable cell lines were obtained. Parasite clones were obtained by limiting dilution.

### Western blot analyses

Transfected *T. cruzi* epimastigotes were harvested separately. Parasites were washed twice in PBS and resuspended in radioimmunoprecipitation assay buffer (RIPA: 150 mM NaCl, 20 mM Tris-HCl [pH 7.5], 1 mM EDTA, 1% SDS, and 0.1% Triton X-100) plus a mammalian cell protease inhibitor mixture (diluted 1:250), 1 mM phenylmethylsulfonyl fluoride, 2.5 mM tosyl phenylalanyl chloromethyl ketone (TPCK), 100 µM N-(*trans*-epoxysuccinyl)-L-leucine 4-guanidinobutylamide (E64), and benzonase nuclease (25 U/mL of culture). The cells were incubated for 1 hour on ice, and the protein concentration was determined by the BCA protein assay. Thirty micrograms of protein from each cell lysate was mixed with 4× Laemmli sample buffer (125 mM Tris-HCl, pH 7, 10% [wt/vol] β-mercaptoethanol, 20% [vol/vol] glycerol, 4.0% [wt/vo]l SDS, and 4.0% [wt/vol] bromophenol blue) before application to 10% SDS-polyacrylamide gels. Separated proteins were transferred onto nitrocellulose membranes with a Bio-Rad Trans-blot apparatus. Membranes were blocked with 5% nonfat dried skim milk in PBS-T (PBS containing 0.1% [vol/vol] Tween 20) overnight at 4°C. Next, membranes were incubated for 1 hour, at RT, with a primary antibody, i.e., monoclonal anti-HA (1:1,000), monoclonal anti-c-Myc-tag (1:100), or monoclonal anti-tubulin (1:20,000). After three washes with PBST, blots were incubated with the appropriate secondary antibody for 1 hour, at RT, in the dark, i.e., IRDye 680RD-conjugated goat anti-rabbit IgG (1:10,000) or IRDye 800CW-conjugated goat anti-mouse IgG (1:10,000). Blots were washed three times with PBST, and Western blot images were obtained and processed with the Odyssey infrared imaging system (LI-COR Biosciences).

### Immunofluorescence assays

*T. cruzi* epimastigotes were washed with PBS and fixed with 4% paraformaldehyde in PBS for 1 hour, at RT. To determine mitochondrial localization of ISC 1 proteins, epimastigotes were incubated with 100  nM MitoTracker deep red FM for 30  minutes at 28°C in the culture medium before the fixing procedure. Cells were allowed to adhere to poly-L-lysine-coated coverslips and then permeabilized for 5  minutes with 0.1% Triton X-100. Permeabilized cells were blocked with PBS containing 3% BSA, 1% fish gelatin, 50  mM NH_4_Cl, and 5% goat serum overnight at 4°C. Then, cells were incubated with a primary antibody (monoclonal anti-HA [1:50] or monoclonal anti-c-Myc-tag [1:10]), diluted in 1% BSA in PBS (pH 8.0) for 1  hour, at RT. Rabbit anti-TbBiP antibodies were used at a dilution of 1:500. Cells were washed three times with 1% BSA in PBS (pH 8.0) and then incubated for 1  hour, at RT, in the dark with Alexa Fluor 488- or Alexa Fluor 546-conjugated goat anti-mouse secondary antibodies (1:1000).

Immunofluorescence of yeast was performed as described (71), with some modifications. Briefly, mid- to late-log phase yeast cells were centrifuged at 700 × *g* for 5 minutes and fixed with 4% paraformaldehyde in SC-URA on a shaker (200 rpm) at 30°C for 1 hour. Cells were collected by centrifugation, washed once with 1 mL of the fresh medium, and incubated with DET (100 mM DTT, 20 mM EDTA, 20 mM Tris-HCl, pH 8.0) at RT for 5 minutes. After collecting the cells by centrifugation, the cell pellet was suspended in 1 mL of 0.9 M sorbitol/PBS (pH 7.4), 20 mg/mL zymolyase was added to make a final concentration of 100 µg/mL, and then incubated on a shaker (200 rpm) for 30–60 minutes at 37°C until cell walls were digested. Spheroplasts were washed gently with 0.9 M sorbitol/PBS, allowed to adhere to poly-L-lysine-coated coverslips, and permeabilized with 1% Triton X-100/0.9 M sorbitol/PBS (pH 7.4) for 10 minutes at RT. After blocking with PEM (100 mM PIPES [pH 7.0], 1 mM EGTA, 0.1 mM MgSO_4_, 1% BSA, and 0.1% NaN_3_) for 1 hour, spheroplasts were labeled in PEM with the rabbit polyclonal glutathione-S-transferase (GST) antibody (1:200) for 1 hour. After thoroughly washing with PEM, cells were incubated with Alexa 488-conjugated goat anti-mouse antibody (1:1,000) for 1 hour, at RT, in the dark.

After labeled with primary and secondary antibodies, the trypanosome or yeast cells were washed and mounted on slides using Fluoromount-G mounting medium containing 5  µg/mL of 4,6-diamidino-2-phenylindole (DAPI) to stain DNA. Differential interference contrast and fluorescence optical images were captured with a 100× objective (1.35-aperture) lens under nonsaturating conditions with an Olympus IX-71 inverted fluorescence microscope with a Photometrix CoolSnapHQ charge-coupled device camera driven by DeltaVision software (Applied Precision, Issaquah, WA). Colocalization analyses were done using FIJI software (ImageJ, National Institutes of Health, Bethesda, MD, USA) with JACoP plugin, where Pearson’s correlation coefficients were obtained.

### Southern blot analysis of *TcPI-PLC1*-KO cells

Two strategies were designed: (i) to confirm the *TcPI-PLC1* deletion and (ii) to confirm the blasticidin insertion in the *TcPI-PLC1-*KO parasites. In the first strategy, genomic DNA from WT and *TcPI-PLC1*-KO epimastigotes was isolated by phenol–chloroform extraction, digested with BamHI, separated on a 0.8% agarose gel, transferred to a nylon membrane, and hybridized with a ^32^P-labeled fragment of 439 nt (*TcPI-PLC1* [nt +1 to +439] obtained by PCR (Table S1, primers 6 and 7) using the cloned *TcPI-PLC1* gene as a template and labeled using [α-^32^P]dCTP (Perkin–Elmer) with random hexanucleotide primers and the Klenow fragment of DNA polymerase (Prim-A-Gene Labeling System). Following hybridization and post-hybridization washes, detection was performed with a phosphor screen.

In the second strategy, genomic DNA from WT and *TcPI-PLC1*-KO epimastigotes was isolated by phenol–chloroform extraction, digested with PvuII, separated on a 0.8% agarose gel, transferred to a nylon membrane, and hybridized with a ^32^P-labeled fragment of 430 nt (*TcPI-PLC1* [nt −503 to −1] obtained by PCR (Table S1, primers 4 and 5) using WT genomic DNA as the template and labeled using [α-^32^P]dCTP (Perkin–Elmer) with random hexanucleotide primers and the Klenow fragment of DNA polymerase (Prim-A-Gene Labeling System). Following hybridization and post-hybridization washes, detection was performed with a phosphor screen.

### Southern blot analysis of *TcITPK1*-SKO cells

Genomic DNA from WT and *TcITPK1*-SKO epimastigotes was isolated by phenol–chloroform extraction, digested with PvuII, separated on a 0.8% agarose gel, and transferred to the nylon membrane and hybridized with a biotin-labeled fragment of 455 nt (*TcITPK1* [nt −729 to −275]) obtained by PCR (Table S1, primers 21 and 22) using WT genomic DNA as the template. The probe was labeled using the Invitrogen BioPrime DNA Labeling System kit. Hybridization was carried out in 0.5 M Na_2_HPO_4_, pH 7.2, and 7% SDS, at 65°C for 18 hours. Post-hybridization washes and detection were performed with the Thermo Scientific Chemiluminescent Nucleic Acid Detection Module kit, following the manufacturer’s recommendations. Signal detection was performed using a ChemiDoc Imaging System (Bio-Rad).

### Southern blot analysis of *TcISC1*-KO cells

Genomic DNA from WT and *TcISC1*-KO epimastigotes was isolated by phenol–chloroform extraction, digested with PvuII, separated on a 0.8% agarose gel, and transferred to the nylon membrane and hybridized with a biotin-labeled fragment of 435 nt (*TcISC1* [nt +694 to +1,128]) obtained by PCR (Table S1, primers 40 and 41) using the cloned *TcISC1* gene as the template. The probe was labeled using the Invitrogen BioPrime DNA Labeling System kit. Hybridization was carried out in 0.5 M Na_2_HPO_4_, pH 7.2, and 7% SDS, at 65°C for 18 hours. Post-hybridization washes and detection were performed with the Thermo Scientific Chemiluminescent Nucleic Acid Detection Module kit, following the manufacturer’s recommendations. Signal detection was performed using a ChemiDoc Imaging System (Bio-Rad).

### *In vitro* metacyclogenesis

We followed the protocol described by Bourguignon et al. (72) with minor modifications. Epimastigotes were obtained after 4 days of incubation in the LIT medium and submitted to a stress (incubation for 2 hours in a medium containing 190 mM NaCl, 17 mM KCl, 2 mM MgCl_2_, 2 mM CaCl_2_, 0.035% sodium bicarbonate, 8 mM phosphate, pH 6.9, at RT; triatome artificial urine [TAU] medium). After this stress, parasites were incubated for 96 hours in the TAU 3AAG medium (which consists of the above-described TAU medium supplemented with 10 mM L-proline, 50 mM sodium L-glutamate, 2 mM sodium L-aspartate, and 10 mM glucose). Cells in the supernatant were collected and fixed with 4% paraformaldehyde in PBS for 1 hour at RT. Cells could adhere to poly-L-lysine-coated coverslips for 20 minutes at RT. Then, cells were washed and mounted on slides using the Fluoromount-G mounting medium containing 5 µg/mL of 2-(4-aminophenyl)-1-indole-6-carboxamidine (DAPI) to stain DNA.

### *In vitro* infection assay

Gamma-irradiated (2,000 rad) Vero cells (4 × 10^5^ cells) were plated onto sterile coverslips in a 12-well plate and incubated overnight at 35°C, 7% CO_2_, in RPMI medium plus 10% fresh fetal bovine serum. Tissue culture-derived trypomastigote collections were incubated at 4°C overnight to allow amastigotes to settle from swimming trypomastigotes. Trypomastigotes from the supernatants of these collections were counted and used to infect the coverslips at a ratio of 50 parasites to one host cell. At 4 hours post-infection, coverslips were washed extensively with Dulbecco’s Hanks’ solution, followed by washing with phosphate-buffered saline (PBS), pH 7.4, to remove any extracellular parasites. Coverslips were fixed immediately in 4% paraformaldehyde in PBS, pH 7.4, at 4°C for 30 minutes. Coverslips were washed once with PBS and mounted onto glass slides in Fluoromount G containing 15 µg/mL of 2-(4-aminophenyl)-1H-indole-6-carboxamidine (DAPI), which stains host and parasite DNA. Coverslips were viewed on an Olympus BX60 microscope to quantify the number of host cells that contained intracellular parasites and the number of intracellular parasites per cell in randomly selected fields. To quantify amastigote replication, the following modifications were used: host cells were infected at a ratio of 10 parasites to one host cell, and coverslips were allowed to incubate for 48 hours post-infection at 35°C, 7% CO_2_, prior to fixation and DAPI staining.

### Inositol phosphate extraction using titanium dioxide beads followed by capillary electrophoresis electrospray ionization mass spectrometry

*T. cruzi* epimastigotes (1 × 10^9^ cells) were harvested and washed twice in washing buffer A with glucose (BAG; 116 mM NaCl, 5.4 mM KCl, 0.8 mM MgSO_4_, 5.5 mM D-glucose, and 50 mM HEPES, pH 7.0). The pellet was then mixed with 1 M perchloric acid, resuspended by sonication (40% amplitude) for 10 seconds and kept on ice for 15 minutes. The samples were centrifuged at 18,000 × *g* for 5 minutes at 4°C, and the supernatants were transferred to new tubes. Three milligrams of TiO_2_ beads (Titansphere TiO 5  µm; GL Sciences) was washed with water and 1 M perchloric acid and added to the samples and left rotating for 30 minutes at 4°C. Beads were centrifuged at 3,500 × *g,* and inositol phosphates were eluted with 2.8% ammonium hydroxide. The ammonia was removed, and the samples were concentrated using a SpeedVac evaporator for 1–3  hours at 45°C. CE-ESI-MS analyses were performed on a bare-fused silica capillary with a length of 100  cm (50  µm internal diameter and 365  µm outer diameter) on an Agilent 7100 capillary electrophoresis system coupled to a Q-TOF (6520, Agilent) equipped with a commercial CE-MS adapter and sprayer kit from Agilent, as described before (24). Data were collected with Agilent OpenLAB CDS Chemstation 2.3.53 and Agilent MassHunter Workstation Acquisition for Q-TOF B.04.00.

### Inositol phosphate extraction using titanium dioxide beads followed by phytase treatment

*T. cruzi* epimastigotes (2 × 10^9^ cells) were harvested and washed twice in washing buffer A with glucose (BAG; 116 mM NaCl, 5.4 mM KCl, 0.8 mM MgSO_4_, 5.5 mM D-glucose and 50 mM HEPES, pH 7.0). The pellet was then mixed with 1 M perchloric acid, resuspended by sonication (40% amplitude) for 10 seconds, and kept at RT for 15 minutes. The samples were centrifuged at 18,000 × *g* for 5 minutes, and the supernatants were transferred to new tubes and boiled for 30 minutes to remove the large amount of polyphosphates present in *T. cruzi*. Five milligrams of TiO_2_ beads (Titansphere TiO 5  µm; GL Sciences) was washed with water and 1 M perchloric acid and added to the sample and left rotating for 30 minutes at RT. Beads were centrifuged at 3,500 × *g,* and inositol phosphates were eluted with 2.8% ammonium hydroxide. The samples were neutralized with perchloric acid and split into two. One half was digested with phytase (0.1 mg/mL) in the same medium at pH 5.0 for 1 hour at 37°C. Samples were mixed with orange G loading buffers and resolved by PAGE using 35% acrylamide/bisacrylamide 19:1 gel in Tris/borate/EDTA (TBE) buffer, as described by Losito et al*.* (73). Gels were stained for 30 minutes, at RT, in the toluidine blue staining solution (20% methanol; 2% glycerol; 0.05% toluidine blue) and then destained for 2 hours with several changes of the same solution without dye. Pictures were taken after exposing the gel on a white light transilluminator. Densitometric analyses were performed with ImageJ software.

### Stable isotope labeling using ^13^C-glucose

Metabolic tracing experiments using ^13^C-glucose were performed in the scrambled cell line (control) and *TcPI-PLC1*-KO mutants. For this, 100 mL of parasite cultures (initial density 2 × 10^6^ cells/mL) was cultivated for 48 hours in the LIT medium without added D-glucose (hereafter referred to as low-glucose LIT) and supplemented with 10% FBS. After this period, samples were harvested by centrifugation (1,600 × *g* for 10 minutes). Then, samples were split into two flasks. One flask was transferred to the fresh LIT medium supplemented with 5 mM ¹³C-glucose and 10% FCS, while the other was supplemented with 5 mM D-glucose and 10% FCS. Both flasks were incubated for an additional 24 hours. Subsequently, the cells were harvested by centrifugation for inositol phosphate extraction and LC-MS analysis.

### Yeast transformation and culture

*S. cerevisiae* strains described before (17) with different genetic backgrounds: wild-type (BY4741), *PLC1*-ablated (*plc1*Δ), and *PLC1-* and *ISC1*-ablated (*plc1*Δ *isc1*Δ) were incubated in 3 mL YPD media at 30°C with shaking at 200 rpm for 8 hours. Fifty microliters of the culture was inoculated in a fresh flask containing 50 mL YPD and incubated at 30°C with shaking at 200 rpm for 16–20 hours. When an OD_600_ of 0.15–0.3 was reached, yeast cells were spun down, and the supernatant was discarded. Cells were resuspended in 100 mL YPD media and incubated for 3–5 hours. Then, cells were washed once with sterile deionized water and resuspended in 3 mL 1.1× TE/LiAc solution and split between two microcentrifuge tubes. Spun-down pellets were resuspended in 600 µL 1.1× TE/LiAc solution. In a new tube, 0.1 mg salmon sperm DNA and 0.1 µg plasmid DNA (pCA45, pCA45-*HsITPK1*, pCA45-*TcITPK1,* pCA45-*TcITPK1-H198A,* or pCA45-*TcITPK1-K242A*) were mixed with 100 µL of competent yeast cells. Cells were then incubated at 30°C for 30 minutes, followed by 42°C in a water bath for 30 minutes, pelleted at 5,000 × *g*, resuspended in water, and plated on selective media. After incubation at 30°C for 3–4 days, colonies were cultured in selective liquid media at 30°C overnight, harvested, and analyzed by plasmid isolation, PCR confirmation (Table S1, primers 23 and 24), and sequencing.

### *myo-*inositol deficient growth assay

Transformed yeast cultures were grown overnight in 5 mL SC-URA media, shaking at 200 rpm, at 30°C. Yeast cultures were adjusted to OD_600_ = 10, and 2 µL was spotted onto SC-URA solid medium with or without *myo-*inositol along with four 10-fold serial dilutions. The plates were incubated at 30°C for 2–5 days and monitored every day. Each assay was repeated three times. For growth assay in liquid media, transformed yeast cultures were grown overnight in 5 mL SC-URA media, with shaking at 200 rpm, at 30°C. Yeast cultures were diluted to OD_600_ = 0.1 in a sterile 96-well assay plate for a total volume of 200 µL in SC-URA media or *myo-*inositol-deficient SC-URA media. For continued measurement of culture OD, the assay plate with lid was placed in the Synergy H1 Hybrid Multi-Mode Microplate Reader (BioTek). Yeast cultures were incubated at 30°C while shaking, and the OD_600_ was collected every 30 minutes for 40 hours. Each assay was repeated three times to achieve biological replicates.

### [^3^H]inositol labeling, IP extraction, and SAX-HPLC analysis

The radioactive isotope labeling, IP extraction, and SAX-HPLC analysis of transformed yeasts were performed as previously described (74). In summary, yeast samples were grown overnight at 30°C in SC-URA media and then used to inoculate a fresh flask of inositol-free SC-URA media containing 5 µCi mL⁻¹ [³H]inositol. This radioactively labeled culture was grown overnight at 30°C, with shaking, to an OD_600_ = 0.5–0.9. Labeled yeasts were collected by centrifugation (2,000 × *g*, 2 minutes, 4°C), washed once with ice-cold water or inositol-free SC-URA media, and resuspended in ice-cold water. Yeast samples were spun down (2,000 × *g*, 2 minutes, 4°C) and resuspended in extraction buffer (1 M perchloric acid, 3 mM EDTA, and 0.1 mg/mL IP_6_) and glass beads. Yeast cell walls were broken by vortexing for 5 minutes at 4°C and debris removed by centrifugation (15,000 × *g*, 5 minutes). The remaining supernatant was neutralized with neutralization buffer (1 M K_2_CO_3_ and 3 mM EDTA) to a pH between 6.0 and 8.0. Tubes were incubated on ice for 2 hours, flicking the mixture every 30 minutes, and then spun-down at (15,000 × *g*, 5 minutes). Supernatants could be stored at 4°C or immediately moved on to SAX-HPLC analysis. Yeast samples were separated onto the PartiSphere SAX (4.6 × 125 mm) column (Hichrom) and eluted with a gradient generated by mixing 1 mM EDTA and Buffer B [1 mM EDTA/1.3 M (NH_4_)_2_HPO_4_, pH 3.8]: 0 to 5 minutes, 0% buffer B; 5 to 10 minutes, 0 to 10% buffer B; 10 to 60 minutes, 10 to 100% buffer B; and 60 to 80 minutes, 100% buffer B. Experiments were done in triplicate.

### Statistical analysis

Statistical analyses were performed with GraphPad Prism software (La Jolla, CA), version 10. Reported values are expressed as means ± S.D of *n* biological experiments, as indicated in the figure legends. The level of significance was evaluated by Student’s *t* test for comparisons between two cell lines, one-way ANOVA for comparisons between more than two cell lines, and two-way ANOVA with multiple comparison tests for analyses of grouped data.

## SUPPLEMENTAL MATERIAL

Supplemental figures mbio.03318-24-s0001.pdf Fig. S1 to S6.

Table S1 mbio.03318-24-s0002.pdf Primers.

ASM does not own the copyrights to Supplemental Material that may be linked to, or accessed through, an article. The authors have granted ASM a non-exclusive, world-wide license to publish the Supplemental Material files. Please contact the corresponding author directly for reuse.
