# Supplementary material for: Generation of inositol polyphosphates through a phospholipase C-independent pathway involving carbohydrate and sphingolipid metabolism in Trypanosoma cruzi
Source: mBio. 2025 Apr 2;16(5):e03318-24. doi: 10.1128/mbio.03318-24 (PMC12077091; doi:10.1128/mbio.03318-24)
Supplement: Supplemental figures — Fig. S1 to S6. [file mbio.03318-24-s0001.pdf]

```
TcITPK1   ------------------------------------MSTAPREKQFVIALCSSERKKPFFEKLQRYVEEKESLEREGDDGAHVTFSFVWMQYDPERNKFSTGTENDADLVLHKVSTLPPK   84
LcIKC     -------------------------------------------------MGKNSKKKLVFSVKANHCIIR-GVV----------------------KKLQ--DDNKIDLVVHDPTQDFFE   46
EhITPK1   -------------------------------------MTTKQTVSLFIWLPESKQKTLFISTKN---HTQFELN------------NIIFDVTLS-TELP--DKEPNA-IITKRTHPVG-   63
OsITPK1   MRVHEEASEDKEREVEEAPDLMPLSPPLTAAATAAVVAVAGQRLVVGYALTKKKVKSFLQPKLLSLARKK-SIH------------FV--SIDET-RPLS--EQGPFDIILHKLTDKE--   100
ZmITPK1   -------------------------MASDAAAEPSSGVTHPPRYVIGYALAPKKQQSFIQPSLVAQAASR-GMD------------LV--PVDAS-QPLA--EQGPFHLLIHKLYGDD--   75
MbITPK1   -------------------------------------------------------------------------------------------MALQ-TQL---EKMGAKVLIHKLTDQLAR   25
DdITPK1   ---------------------------------MGRNNTLPNDFTVGYYLSDSKINKLKWNLFVDMCKEKYNIN------------VV--PIDMN-KDINSINTRPYHVIIDKLTDELGD   72
DrITPK1   ------------------------------------MQTFVKGKRVGYWLSEKKIKKLNFQTFVDLCRKQ-GIE------------MI--QLDLS-QPIE--SQGPFDVIIHKLTDHIVD   66
GgITPK1   ------------------------------------MQTFLKGKRVGYWLSEKKIRKLNFQAFAELCRKR-GVE------------VV--QLDLT-KPIE--DQGPLDVIIHKLTDVILE   66
HsITPK1   ------------------------------------MQTFLKGKRVGYWLSEKKIKKLNFQAFAELCRKR-GME------------VV--QLNLS-RPIE--EQGPLDVIIHKLTDVILE   66
MmITPK1   ------------------------------------MQTFLKGKRVGYWLSEKKVKKLNFQAFAELCRKR-GIE------------VV--QLNLS-RPIE--EQGPLDVIIHKLTDVILE   66
                                                                                                            :          :: .

TcITPK1   AV---GALCRWCAAASRRRRRGRIPPVIIIDPVELTRLVLQRSLLYKMLDGR--------------LKRPLCPVPHSWLWIRDGASLTPLGLSSFVLSDEEDTEVMGASQS-SRETWWIV   186
LcIKC     LETIPQFLE-DIDLLVVKVRNDCSIDLLHLAKIYKIPTLHNFDVVSTCKNKISLDYTLRKIFNDNSKKLSKFMLPKSWNHSL--------------------MDVSRFKKWASTRLPIVI   145
EhITPK1   ---------KMADEMR-KYE-KDHPKVLFLESSAIHDMMSSREEINALLIKNNI------PI-------PNSFSVKS----K--------------------EEVIQLLQSKQLILPFIV   135
OsITPK1   ----------WQQVLE-DYR-EEHPEVTVLDPPNAIQHLHNRQSMLQEVADLNL------SNAYGEVCTPRQLVIMK-DPLS----------------------IPSAVAKAGLTLPLVA   179
ZmITPK1   ----------WRAQLV-AFA-ARHPAVPIVDPPHAIDRLHNRISMLQVVSELDH------AADQDSTFGIPSQVVVY-DAAA----------------------LADFGLLAALRFPLIA   154
MbITPK1   LEQGDTTAETLVHQLE-AWT-A-SGSQRVLDPLPRVRPLVDRGATLGLLEQLSS------QLPDGFAIPPSRVAHSA-P-------------------------QAAGWLTSALRWPLIV   110
DdITPK1   LDNPS--NKQKVDYIQ-SLI-DKFPSIVEVDPLESQKPVLSRDTLTKLLDKLND------VSPELNIKNPKFVLVPE-DYNN--------------------NDYNQLLKDANIKFPVVC   161
DrITPK1   ADQNVTESLLLVQGVQ-DYI-DAHPETVILDPLPAIRTLLDRCKSYKLIHKLEH------SMEDDRICSPPFMVLKT-ECGF---------------------ETLEQLHKHGITFPFIC   156
GgITPK1   ADQNDSQSLELVQRFQ-EYI-DAHPETIILDPLPAIRTLLDRSKSYELIRQIEA------YMQDERICSPPFMELTS-ACGE---------------------DTLQLIEKNGLAFPFIC   156
HsITPK1   ADQNDSQSLELVHRFQ-EYI-DAHPETIVLDPLPAIRTLLDRSKSYELIRKIEA------YMEDDRICSPPFMELTS-LCGD---------------------DTMRLLEKNGLTFPFIC   156
MmITPK1   ADQNDSQSLELVHRFQ-EYI-DAHPETIVLDPLPAIRTLLDRSKSYELIRKIEA------YMKDDRICSPPFMELTS-LCGE---------------------DTMRLLEQNGLAFPFIC   156
                                 :        : .                                                                             :

TcITPK1   KSDISTGPSFTHQMVIWKGCRPEKSLPEEVLSLLSSCVNSFVLQEFFLHAISVVIKVYCIGTVVFVKAVPTAPLLRCVLSKMGGPVFVDSQ---EKFPIDAGWVEEEARWR------NY-   296
LcIKC     KSHNQHDKYNRFNFLVQKIDEVDKF-CEKYKN---FLYYDVYIQKFIE-CDGFERKVYVIGDKVFGIIRENPIYIYLR---------------EKPKNIDVGLIKR--------------   231
EhITPK1   KPENAQGTFNAHQMKI-------VL-EQEGID---DIHFPCLCQHYIN-HNNKIVKVFCIGNTLKWQTRTSLPNVHRCGIK---SVDFNNQHLEDILSWPEGVIDKQDIIENSANRFGSK   240
OsITPK1   KPLVVDGTSKSHELSL-------AY-VETSLS---MLDPPLVLQEFVN-HGGILFKVYVVGETIRVVRRFSLPDVNIYDLENNDGIF-RFPRVSCATN--------------TAED----   268
ZmITPK1   KPLVADGTAKSHKMSL-------VY-HREGLG---KLRPPLVLQEFVN-HGGVIFKVYVVGGHVTCVKRRSLPDVSPEDDASAQGSV-SFSQVSNLPT--------------ERTAEEYY   247
MbITPK1   KPLPAHGSSDAHRMCI-------VN-NLACLE---QLSYPVLCQNFYD-HDALLYKVFVLGDRHHVVPRRPSIRNVDPAARGEATLMAFDSHDVSKAHS--------------QTYLNDAV   204
DdITPK1   KTIKACGSKESHYMGI-------VF-NEKDIH---QFKQPMLIQEFIN-HNAIIYKVFAIGDFIQVVHRKSIRNMNENE-N--ELIKFDSQ---KPFP--------------TSL-LPTD   248
DrITPK1   KPQVAHGT-NSHEMAI-------IF-SEEDLK---DIKPPCVLQSFIN-HNAVLYKVFVVGEAYSVVQRPSIRNFPSGP-TDRRAISFNSHHVSKPES--------------SSHLTCRD   248
GgITPK1   KTRVAHGT-NSHEMAI-------IF-NQEGLK---AVRPPCVIQSFIN-HNAVLYKVFVVGESYTVVKRPSLKNFSAGI-SDRESIFFNSHNVSKPES--------------SSVLTALD   248
HsITPK1   KTRVAHGT-NSHEMAI-------VF-NQEGLN---AIQPPCVVQNFIN-HNAVLYKVFVVGESYTVVQRPSLKNFSAGT-SDRESIFFNSHNVSKPES--------------SSVLTELD   248
MmITPK1   KTRVAHGT-NSHEMAI-------VF-NQEGLN---AIQPPCVVQNFIN-HNAVLYKVFVVGESYTVVQRPSLKNFSAGT-SDRESIFFNSHNVSKPES--------------SSVLTELD   248
          *     .    . : :                           * :         **: :*         .   .

TcITPK1   -----LAVGGRAYTQCSQIAEQLVRELGLTLFGFDLLLVPKKVSGQCDTPLGASLRDAPLFDEVTGAPSALLCSATPVIVDVNYFPGFSGVENVAEHVLDVIKSKALGTPVSKRS-----   406
LcIKC     -------ERFKVTEDILQLSKILSHDLDLKIFGFDLIQPINQ-------------------------------NK-LFLVDLNDFPSFKGIPNIENEMKNYIKNYVLAL-----------   301
EhITPK1   ILEDPILLNLTSEAEMRDLAYKVRCALGVQLCGIDFIKENEQ-------------------------------GN-PLVVDVNVFPSYGGKVDFDWFVEKV-ALCYTEVAKI--------   319
OsITPK1   AEVDPSIAELPPKPLLEKLGRELRRRLGLRLFNFDMIREHGR-------------------------------KDRYYVIDINYFPGYGKMPGYEHIFIDFLLSLVQNKYKRRLSGS---   354
ZmITPK1   GEKSLEDAVVPPAAFINQIAGGLRRALGLQLFNFDMIRDVRA-------------------------------GDRYLVIDINYFPGYAKMPGYETVLTDFFWEMVHKDGVGNQQEEKGA   336
MbITPK1   AMKTALASNVLRRPDLALLADRMRQHLGLTLFGFDVA--AET-------------------------------SV-HYIVDVNYFPGYKGIESFQEDLCHLLRQTVDSLE----------   280
DdITPK1   GQELKI--EMPSKSTLSVISKDIQKNLDISLFGFDVIVDCET-------------------------------KK-LAVVDINYFPTFSGVDDFYTLLIEHVINVYKRKSSV--------   326
DrITPK1   NMVGQS--WKPSNEVIQKISRKLHQALGISLFGIDIIINNQT-------------------------------GQ-HAVIDINAFPGYEGVPEFFDDLLSHISSVLQGQVCN----GVAC   330
GgITPK1   KIEGVF--ERPDDDVIREISKALRQALGVSLFGIDIIINNQT-------------------------------GQ-HAVIDINAFPGYEGVSEFFTDLLNHIAAVLQGQAP---------   325
HsITPK1   KIEGVF--ERPSDEVIRELSRALRQALGVSLFGIDIIINNQT-------------------------------GQ-HAVIDINAFPGYEGVSEFFTDLLNHIATVLQGQSTA----MAAT   330
MmITPK1   KIEGVF--ERPSDEVIRELSRALRQALGVSLFGIDIIINNQT-------------------------------GQ-HAVIDVNAFPGYEGVSEFFTDLLNHIATVLQGQSTG----GAAT   330
                            :.  :   *.: : .:*.                                          ::*:* ** :         .

TcITPK1   -------------STEGKLFNGNFCC----------------------------------------------------------------   419
LcIKC     ------------------------------------------------------------------------------------------   301
EhITPK1   ------------------------------------------------------------------------------------------   319
OsITPK1   ------------------------------------------------------------------------------------------   354
ZmITPK1   NHVVVK------------------------------------------------------------------------------------   342
MbITPK1   ------------------------------------------------------------------------------------------   280
DdITPK1   ------------------------------------------------------------------------------------------   326
DrITPK1   GHLRVNG----------------TA-QSQTVHCGMLGNDSSWLMDSEGIKK-------GPHQGLSCCGGCMTPNFHQHTRSSLTAETSSQ   396
GgITPK1   -EVTQLNRSKLLAEQTGGIMDERICCASTGCI-SVMGKDSSWIVESET-----NSSVKLQHQRLGC-NSAVSPSFQQHCVATLATKASSQ   407
HsITPK1   GDVALLRHSKLLAEPAGGLVGERTCSASPGCCGSMMGQDAPWKAEADA-----GGTAKLPHQRLGC-NAGVSPSFQQHCVASLATKASSQ   414
MmITPK1   EEVAPLRHNRLLAEPAGSLAGERTCSASPGCCGSMKGQDTPWKTETEAGNMGAGASAKLPHQRLGC-TTGVSPSFQQHCVASLATKASSQ   419
```

Figure S1. Clustal Omega Multiple Sequence Alignment of ITPK1 Orthologs with TcITPK1 protein. Tc: *Trypanosoma cruzi* (KAF8297109.1), Lc: *Lokiarchaeum. Candidatus* (KKK40176.1), Eh: *Entamoeba histolytica* (XP_651704), Os: *Oryza sativa* (NP_001409892), Zm: *Zea mays* (NP_001105901), Mb: *Monosiga brevicollis* (XP_001747651), Dd: *Dictyostelium discoideum* (XP_646244), Dr: *Danio rerio* (XP_009298398), Gg: *Gallus gallus* (046774577), Hs: *Homo sapiens* (NP_001136065), Mm: *Mus musculus* (NP_766172). Pink highlighted amino acid residues denote highly evolutionarily conserved IP and ATP binding sites of ITPK1. Yellow highlighted amino acid residues denote less evolutionarily conserved IP and ATP binding sites of ITPK1.

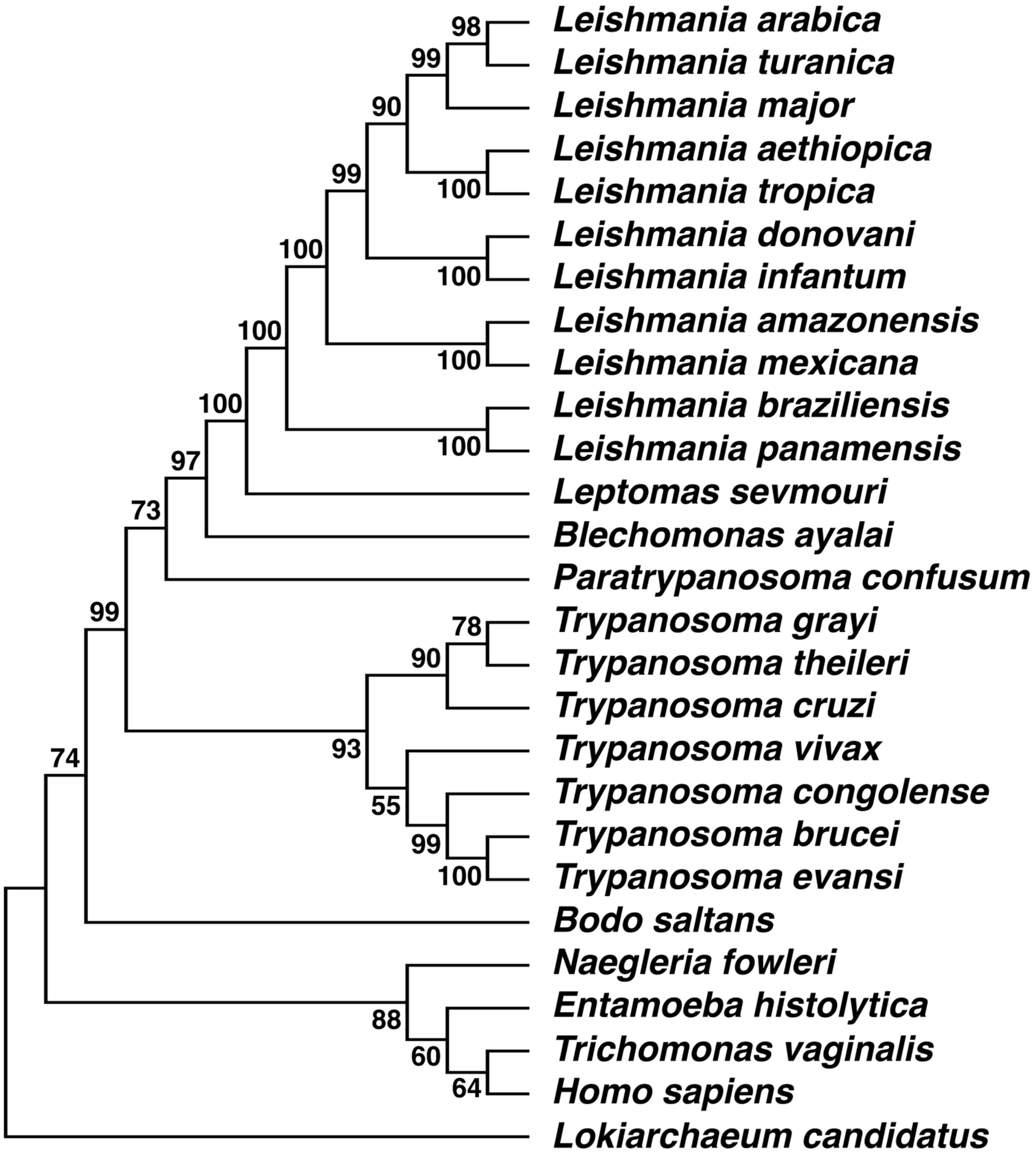

Figure S2. Phylogenetic tree of ITPK1 homologs in Protist Parasites. ITPK1 phylogenetic tree was created using orthologs identified in TriTrypDB and previously published manuscripts using reference strains and selected non-reference strains pertinent to the protein of interest (TcYC6_0083580) with the neighbor-joining method. Branches corresponding to partitions reproduced in less than 50% bootstrap replicates are collapsed. The percentage of replicate trees in which the associated taxa clustered together in the bootstrap test (1000 replicates) are shown next to the branches. The evolutionary distances were computed using the JTT matrix-based method and are in the units of the number of amino acid substitutions per site. The rate variation among sites was modeled with a gamma distribution (shape parameter = 4). This analysis involved 27 amino acid sequences. All ambiguous positions were removed for each sequence pair (pairwise deletion option). There was a total of 773 positions in the final dataset. Evolutionary analyses were conducted in MEGA11. GenBank or TriTrypDB accession numbers of sequences used are: *Leishmania arabica* (LARLEM1108_240026100.1), *Leishmania turanica* (LTULEM423_240026300), *Leishmania major* (XP_001683711.1), *Leishmania aethiopica* (LAEL147_000395100), *Leishmania tropica* (LTRL590_240027000),

Leishmania donovani (LdBPK_242010.1), *Leishmania infantum* (XP_001465992.1), *Leishmania amazonensis* (LAMA_000473700), *Leishmania mexicana* (XP_003876011.1), *Leishmania braziliensis* (KAI5688245), *Leishmania panamensis* (XP_010699530.1), *Leptomas seymouri* (Lsey_0265_0130), *Blechomonas ayalai* (Baya_059_0260), *Paratrypanosoma confusum* (PCON_0066560), *Trypanosoma brucei* (XP_847459), *Trypanosoma evansi* (TevSTIB805.8.6580), *Trypanosoma congolense* (TcIL3000_8_6140), *Trypanosoma vivax* (TvY486_0805890), *Trypanosoma grayi* (XP_009312317.1), *Trypanosoma theileri* (XP_028882908.1), *Trypanosoma cruzi* (KAF8297109.1), *Bodo saltans* (CUG31837), *Naegleria fowleri* (XP_044569723), *Trichomonas vaginalis* (XP_001325482), *Entamoeba histolytica* (XP_651704), *Homo sapiens* (NP_001136065), and *Lokiarchaeum candidatus* (KKK40176.1).

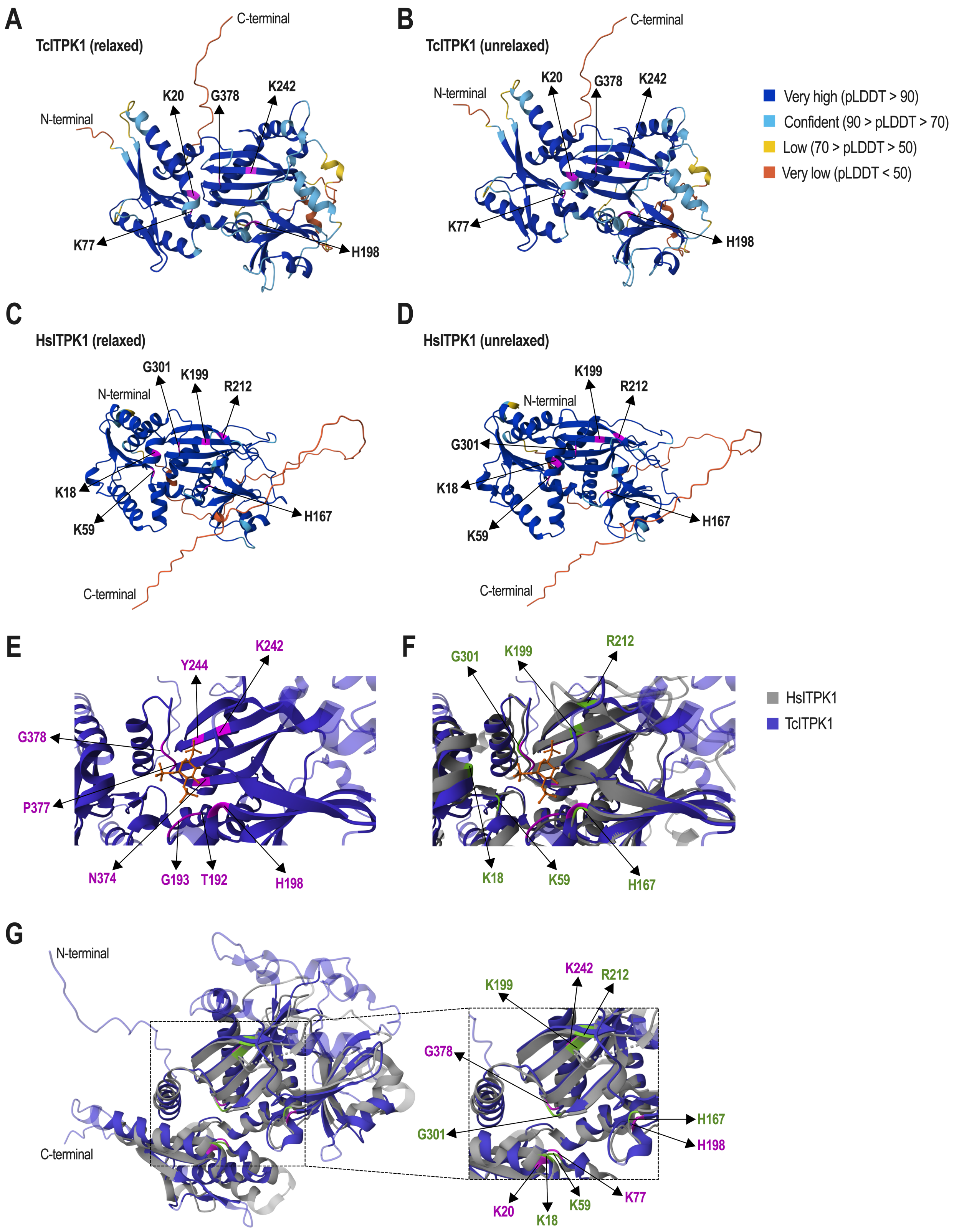

Figure S3. TcITPK1 structural information. (A-B) Structural prediction of TcITPK1 using AlphaFold-2.1.1 for both relaxed (A) and unrelaxed (B) ribbon models generated by iCn3D (NCBI) with AlphaFold Confidence indicator.

Conserved residues are in pink. (C-D) Structural prediction of HsITPK1 using AlphaFold-2.1.1 for both relaxed (C) and unrelaxed (D) ribbon models generated by iCn3D (NCBI) with AlphaFold Confidence indicator. Conserved residues are in pink. (E) Following prediction of the three-dimensional structure of TcITPK1 by AlphaFold-2.1.1, COFACTOR was used to predict ligand-binding sites. IP_3 (orange) was predicted to bind to TcITPK1 at residues K20, T192, G193, H198, K242, Y244, Q277, N374, P377, and G378 (pink). (F) The predicted TcITPK1 model with IP_3 (orange) was structurally aligned with the HsITPK1 model using TM-align parameters on RCSB PDB. Conserved residues are highlighted in green for HsITPK1. TcITPK1 residues that are predicted to interact with IP_3 are shown in pink. (G) Structural comparison of the AlphaFold-predicted TcITPK1 (relaxed) and the X-Ray diffraction-resolved HsITPK1 (PDB: 2ODT) was performed using pairwise structure alignment with the jFATCAT (flexible) parameters on RCSB PDB. Conserved residues are highlighted in green for HsITPK1 and in pink for TcITPK1.

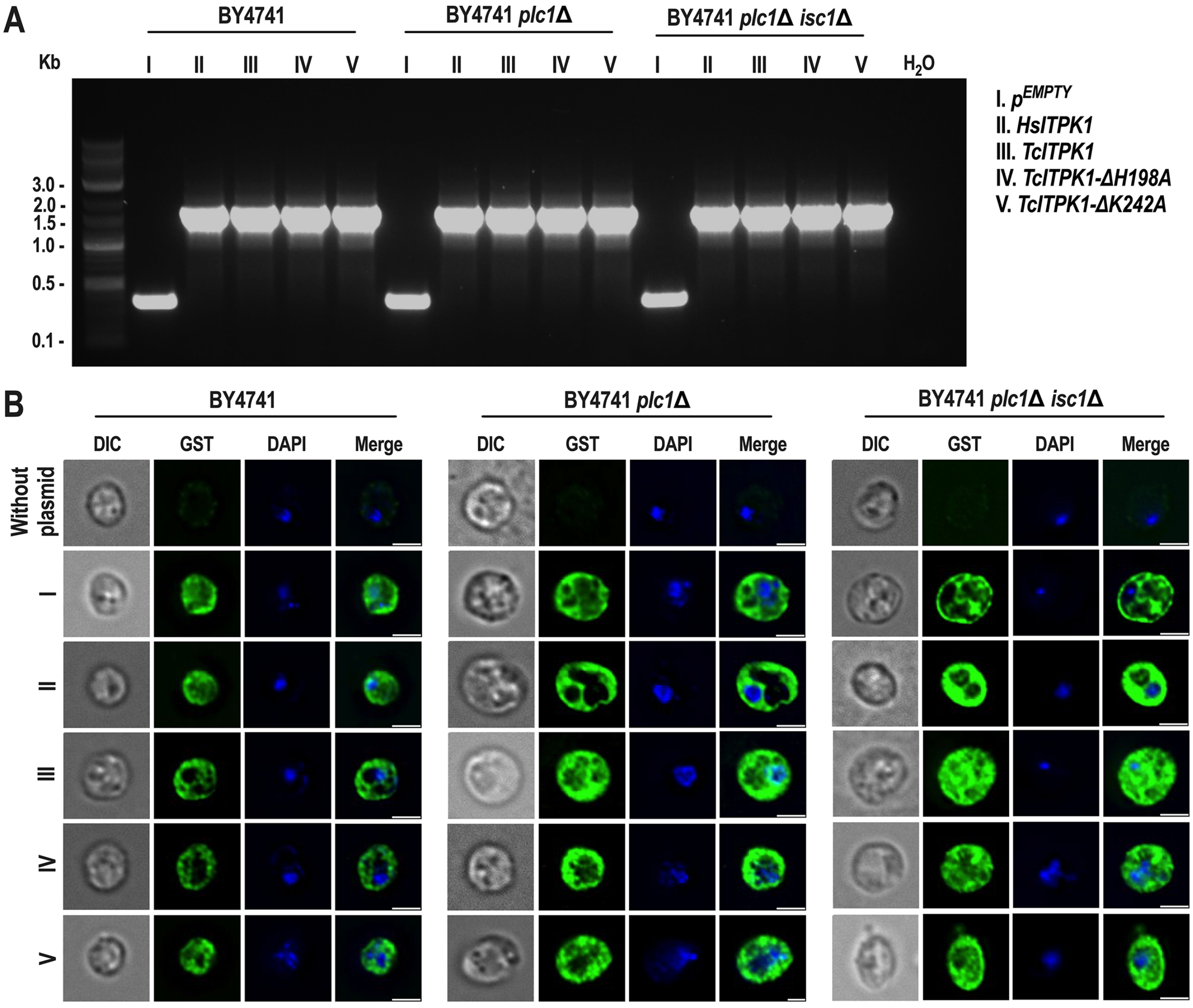

Figure S4. Verification of the successful plasmid incorporation and protein expression in yeast. (A) Plasmids were isolated from *S. cerevisiae* wild type (BY4741), *PLC1*-ablated (*plc1Δ*), and *PLC1* and ISC1-ablated (*plc1Δ isc1Δ*) transformed with pCA45, pCA45-*HsITPK1*, pCA45-*TcITPK1,* pCA45-*TcITPK1-H198A* or pCA45-*TcITPK1-K242A* plasmids. PCR analysis was done using the primers 23 and 24 (Table S1). (B) Expression of HsITPK1, TcITPK1, TcITPK1-H198A, and TcITPK1-K242A was confirmed by immunofluorescence assays using anti-GST antibodies. DIC, differential interference contrast. GST (green), HsITPK1, TcITPK1, TcITPK1-H198A, or TcITPK1-K242A. Merge image shows HsITPK1, TcITPK1, TcITPK1-H198A, or TcITPK1-K242A (green) and DAPI staining (blue).

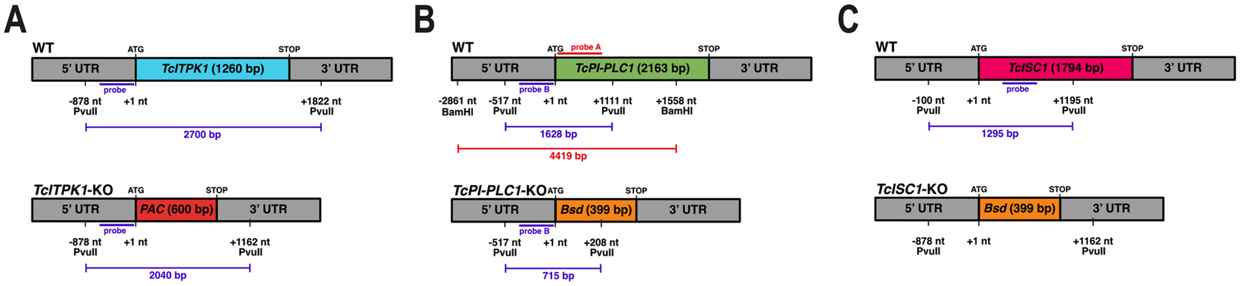

Figure S5. Southern blot strategies. (A) Genomic DNA from wild type and *TcITPK1*-SKO were digested with PvuII restriction enzyme. The blot was hybridized with a biotin-labeled probe corresponding to 455 bp of *TcITPK1* 5' UTR (nt -729 to -275), revealing a 2700 bp band for PvuII-digested gDNA from WT cells, and a 2040 bp band for PvuII-digested gDNA from *TcITPK1*-SKO cells. (B) Genomic DNA from wild type and *TcPI-PLC1*-KO parasites were digested with BamHI restriction enzyme. The blot was hybridized with a with a ^{32}P-labeled probe corresponding to 439 bp (probe A) of *TcPI-PLC1* (nt +1 to +439), revealing a 4419 bp band only for BamHI-digested gDNA from WT cells. When the gDNA from WT and *TcPI-PLC1*-KO parasites were digested with PvuII restriction enzyme, the blot was hybridized with a ^{32}P-labeled probe corresponding to 460 bp (probe B) of *TcPI-PLC1* 5' UTR (nt -503 to -1), revealing a 1628 bp band for PvuII-digested gDNA from WT cells, and a 715 bp band for PvuII-digested gDNA from *TcPI-PLC1*-KO cells. (C) Genomic DNA from wild type and *TcISC1*-KO were digested with PvuII restriction enzyme. The blot was hybridized with a biotin-labeled probe corresponding to 435 bp of *TcISC1* (nt +694 to +1128), revealing a 1295 bp band only for PvuII-digested gDNA from WT cells.

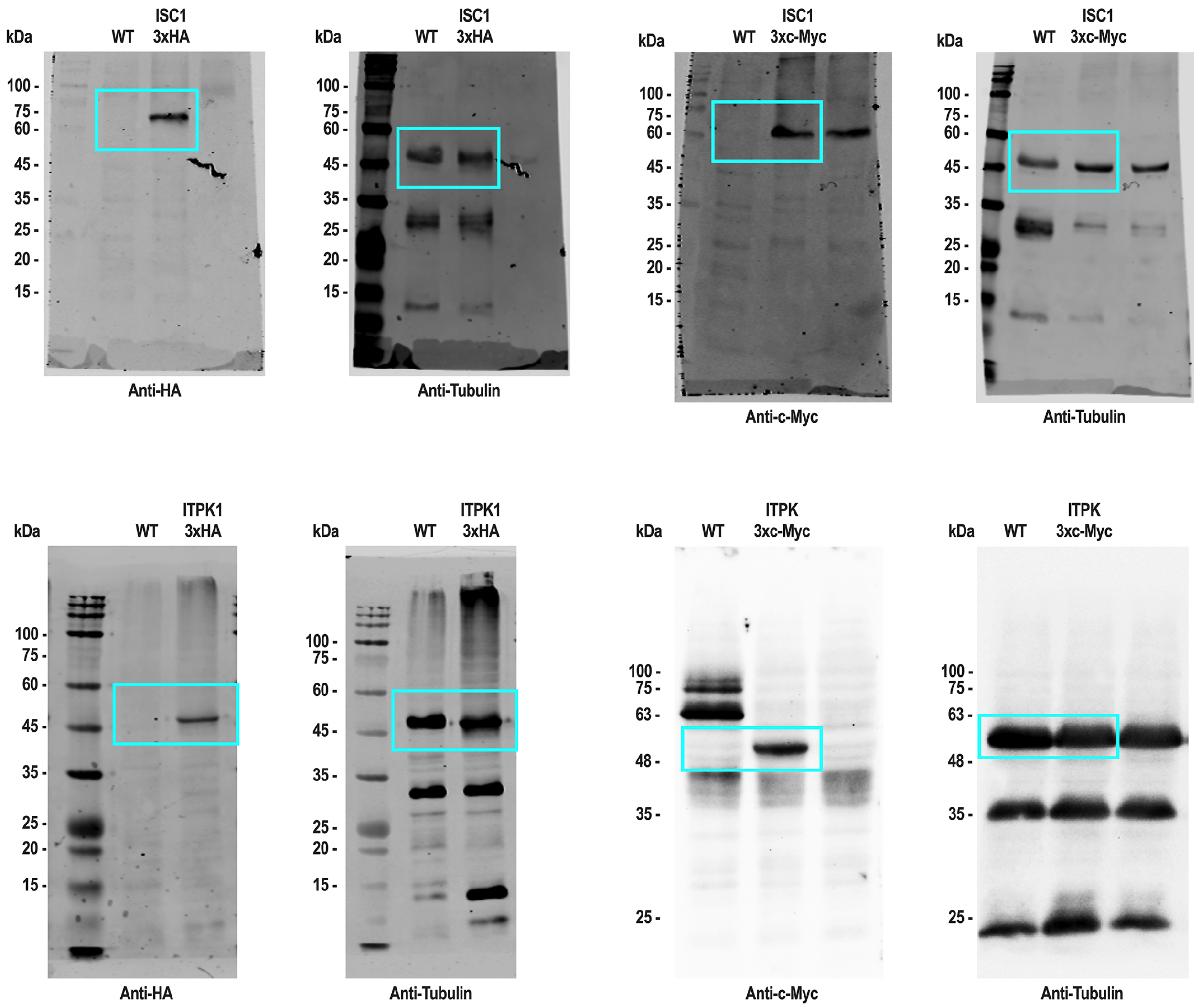

Figure S6. Complete western blots shown in this work.
