# Supplementary material for: Generation of inositol polyphosphates through a phospholipase C-independent pathway involving carbohydrate and sphingolipid metabolism in Trypanosoma cruzi
Source: mBio. 2025 Apr 2;16(5):e03318-24. doi: 10.1128/mbio.03318-24 (PMC12077091; doi:10.1128/mbio.03318-24)
Supplement: Table S1 — Primers. [file mbio.03318-24-s0002.pdf]

N°	Primer name	Sequence (5′ - 3′)
1	FwTcPLC_sgRNA1	GATCGGATCCCGTGATATCCTACTTTGCAGGTTTTAGAGCTAGAAATAGC
2	FwTcPLC_donor	ATTAGGCATAGAAGCGGAAAAGAGTGAAGCGAAAAAGGAAAGGAAATAGGTGAAGCGGGAAAACAACAACAACAACAACAGATTTTGAGGAGGGTTTACAATGGCCAAGCCTTTGTCTCAAG
3	RvTcPLC_donor	CACGCTCCATGCAAATGACGTCAAGAAAAAGTAAGGGAGCATGTGAGAAATGAACCAAAGATGGAATTCCCGTTGATATCATGCAATGGAATTTGTCGAATTAGCCCTCCCACACATAAC
4	Fw_TcPLC_3UTR_probe	AGAGACGCGCAACATACATG
5	Rv_TcPLC_3UTR_probe	TCACTCTTTTCCGCTTCTATGC
6	FwATG-probe_TcPLC	ATGGGTCTTTGTACGAGTAAGTG
7	Rv-probe_TcPLC	TTCCACCGGCAGCCTTTAC
8	FwTcPLC_KO_check	AGGAAAGGAAATAGGTGAAGCG
9	RvTcPLC_KO_check	TCACGCTCCATGCAAATGACG
10	FwTcITPK1_CTag_sgRNA	GATCGGATCCAGATGAAGGGCGAAACATTTGTTTTAGAGCTAGAAATAGC
11	FwTcITPK1_CTag_donor	TCAAAGGCACTGGGTACTCCCGTCTCGAAAAGATCCTCAACTGAGGGAAAACTGTTTAACGGCAACTTCTGCTGCGGTACCGGGCCCCCCCTCGAG
12	RvTcITPK1_CTag_donor	CAGTAGATGCATTTGCTCACACGCATAGAGCATCTTAAAACACACACAAACACACACACACACAGAAAAATGCCTTGGCGGCCGCTCTAGAACTAGTGGAT
13	FwTcITPK1_CTag_check	TGAGCATGTGTTGGATGTCATTAAG
14	RvTcITPK1_CTag_check	GTAGAGATATAACACGGCGTTTACG
15	RvG00_all	AAAAGCACCGACTCGGTGCCACTTTTTCAAGTTGATAACGGACTAGCCTTATTTTAACTTGCTATTTCTAGCTCTAAAAC
16	FwTcITPK1_sgRNA_T7Cas9_KO	GAAATTAATACGACTCACTATAGGTGTGTCTTGTCGAAGATGGGGTTTTAGAGCTAGAAATAGC
17	FwTcITPK1_KO_donor	TTCCCAGCGTGGTGCTGAAGAGGAGAAGCAAACTCTGTACGCCGCGGGAATTCGATTATG
18	RvTcITPK1_KO_donor	GAAAAATGCCTAAATGTTTCGCCCTTCATCTCCACTTCGCGAATTCACTAGTGATTTCAC
19	FwTcITPK1_KO_check	ATTCTCTACTTCCCTTGTATCTTCT
20	RvTcITPK1_KO_check	AGTAGATGCATTTGCTCACA
21	FwTcITPK1_probe	TATAAAGGGACCGTGATGC
22	RvTcITPK1_probe	ATCTCACCACACAACACAA
23	FwpCA45_yeast_check	atgtgcctggatgcgttc
24	RvpCA45_yeast_check	gacataactaattacatgatgcgg
25	FwTcITPK1_H198A	TCCTTCACGGCCCAGATGGTC
26	RvTcITPK1_H198A	GACCATCTGGGCCGTGAAGGA
27	FwTcITPK1_K242A	AGTTGTCATCGCAGTTTACTGCATTGGC
28	RvTcITPK1_K242A	GCCAATGCAGTAAACTGCGATGACAACT
29	FwTcITPK1_SalI	TCACGTCGACAATGAGCACTGCTCCAAGAGG
30	RvTcITPK1_NotI	ATAAGAATGCGGCCGCTTAGCAGCAGAAGTTGCC
31	FwTcITPK1_XbaI	gatcTCTAGAATGAGCACTGCTCCAAGAGAGAAGCAGT
32	RvTcITPK1_XhoI	ctatCTCGAGGCAGCAGAAGTTGCCGTTAAA
33	FwTcISC1_XbaI	gatcTCTAGAATGCCAACTGAAATTACGGT
34	RvTcISC1_XhoI	ctatCTCGAGTCCTTGGCAGGCTTGTTT
35	FwTcISC1_sgRNA_T7Cas9_KO	GAAATTAATACGACTCACTATAGGGTCCTGAGCTTTAACCTGTGGTTTTAGAGCTAGAAATAGC
36	Fw_TcISC1_KO_donor	GAACGAGTCTCCATTCCTCATCTTCAACCACACGGACGGAGCCGCGGGAATTCGATTATG
37	Rv_TcISC1_KO_donor	CTTTTTGTGTTATCACGTGTTCCTCCCCTCCGTTGCCCGCGAATTCACTAGTGATTTCAC
38	FwTcISC1_check	GGATTACGCCGGTATAGC
39	RvTcISC1_check	CAACAACCAACGCTGC
40	FwTcISC1_probe	GAGCTCATTATTTCCAATCTGC
41	RvTcISC1_probe	AAAATCTCCTCCAATAATAACACG
42	FwTcISC1_Ctag_sgRNA	GATCGGATCCGAACACGTGATAACACAAAAGTTTTAGAGCTAGAAATAGC
43	FwTcISC1_Ctag_ultramer	TCTCAGCGCCGCATCCGTCACAGTGGCACTACTTCAGCGGATCGCCAACGCGAAGGTCATGGCGGAGCAAGTTAACATGCTCAAACAAGCCTGCCAAGGAGGTACCGGGCCCCCCCTCGAG
44	RvTcISC1_Ctag_ultramer	AAAGCACAACACAATCGTATAAAACATAATATAAAATCTCATTTTTCAAATCACAACAGTTTTCCTTCTTTTTTTTTTTTTTTTTTTTTTGATTTCTCCTTGGCGGCCGCTCTAGAACTAGTGGAT
45	RvcTag_all	CAGTGGATCCAAAAAAGCACCGACTCGGTG
46	RvHX1	TAATTTCGCTTTCGTGCGTG
47	RvSgRNA	CAGTGGATCCAAAAAAGCACCGACTCGGTG

Table 1: Primers used in this work.
